# Supplementary material for: Mixed-Method Systematic Review and Meta-Analysis of Shared Decision-Making Tools for Cancer Screening
Source: Cancers (Basel). 2023 Jul 29;15(15):3867. doi: 10.3390/cancers15153867 (PMC10417450; doi:10.3390/cancers15153867)
Supplement: Supplementary file 1 [file cancers-15-03867-s001.zip › cancers-2455744-supplementary/Supplementary file_4_certainty of evidence copy.docx]

**Supplementary file 4- Certainty of evidence**

**Table S1.** GRADE Summary findings on the effectiveness of shared decision-making tool for cancer screening to increase informed decision-making

| **Outcome measure**  **Overall**  **Vulnerable vs. Non-vulnerable population**  **Intervention duration** | **Number of participants \|**  **Number of studies** | **Relative effect or Difference**  **(95% CI)** | **Certainty** | **Reasons for such judgement** |
| --- | --- | --- | --- | --- |
| **(Improved) Knowledge on cancer and cancer screening**  Overall | 6,211 \|  15 RCTs | **MD= 13.62**  (8.28 to 18.95) | ⨁⨁⨁⨁  HIGH | No concerns in all domains |
| **Knowledge on cancer and cancer screening**  Vulnerable people | 3, 263 \|  9 RCTs | **MD= 15.44**  (9.40 to 21. 49) | ⨁⨁⨁⨁  HIGH | No concerns in all domains |
| **Knowledge on cancer and cancer screening**  Non-vulnerable people | 2, 120 \|  4 RCTs | **MD= 13.74**  (-10.0 to 37.48) | ⨁⨁⨁  MODERATE | Less precision with wide confidence intervals, and inconsistent results |
| **(Reduced) Decision conflict**  Overall | 4, 830 \|  13 studies | **SMD= -0.71**  (-1.23 to -0.19) | ⨁⨁⨁  MODERATE | Less precision with some studies reporting substantial standard errors |
| **Decision conflict**  Intervention duration: 6 months or less | 1, 838 \|  7 studies | **SMD= -1.17**  (-2.07 to -0.27) | ⨁⨁⨁  MODERATE | Less precision with some studies reporting substantial standard errors |
| **Decision conflict**  Intervention duration: Above 6 months | 2, 231  4 studies | **SMD= -0.19**  (-0.27 to -0.11) | ⨁⨁⨁  MODERATE | Small effect size |
| **Intention to screen**  Overall | 4, 333  13 studies | **RR= 1.11**  (0.95 to 1.30) | ⨁⨁⨁  MODERATE | Inconsistent results |
| **Intention to screen**  Vulnerable people | 3, 028  10 studies | **RR= 1.17**  (1.07 to 1.29) | ⨁⨁⨁⨁  HIGH | No concerns in all domains |
| **Intention to screen**  Non-vulnerable people | 1, 205  4 studies | **RR= 1.03**  (0.44 to 2.43) | ⨁⨁⨁  MODERATE | Small effect size and inconsistent results |
| ***The risk in the intervention group** (and its 95% confidence interval) is based on the assumed risk in the comparison group and the relative effect of the intervention (and its 95% CI)  CI: Confidence interval; RR: Relative risk; SDM: Standardized mean difference; MD: Mean difference  **Domains assessed** included study limitation, imprecision, inconsistency of results, indirectness of evidence, and publication bias (except for decision conflict outcomes) | | | | |
| **GRADE Working Group grades of evidence** ⨁⨁⨁⨁ **High certainty:** We are very confident that the true effect lies close to that of the estimate of the effect  ⨁⨁⨁ **Moderate certainty:** We are moderately confidence in the effect estimate: The true effect is likely to be close to the estimate of the effect, but  there is a possibility that it is substantially different  ⨁⨁ **Low certainty:** Our confidence in the effect estimate is limited: The true effect may be substantially different from the estimate of the effect  ⨁ **Very low certainty:** We have very little confidence in the effect estimate: The true effect is likely to be substantially different from the estimate of  effect | | | | |
|  |  |  |  |  |

**Table S2. GRADE-CERQUAL Approach**

| Study ID  (First author surname, year)  Study design \|  Population eligible in the review | Methodological limitations  Judgement \| Reasons for such judgement | Coherence  Judgement \| Reasons for such judgement | Adequacy  Judgement \| Reasons for such judgement | Relevance  Judgement \| Reasons for such judgement | Overall confidence of the evidence in the individual study  Judgement \| Reasons for such judgement |
| --- | --- | --- | --- | --- | --- |
| Akanuwe 2020 (1) | No concerns  Although the sample size was small, the authors mentioned that the data was sufficient to achieve data saturation. They also mentioned the conduct of purposive sampling strategy to recruit participants until they achieved diversity in the sample in terms of age, gender, ethnicity and cancer risk. Multiple researchers were also involved in the data collection and analysis. | Minor concerns    The study did not explicitly investigate service users' preferences regarding particular shared decision-making (SDM) tools or methods. Nonetheless, the study findings underscored the significance of customizing cancer risk communication to the individual. | No concerns  The data gathered from the study participants yielded a comprehensive to support study findings. The rich description of the data collected to support their study findings was also observed based on the direct quotes from the participants. | No concerns  The study carries significant relevance for the advancement and integration of shared decision-making (SDM) tools in cancer risk communication. The results hold substantial implications for enhancing the quality and efficacy of cancer risk communication practices. | High confidence  Only minor concerns on the methodological limitation and coherence were observed. |
| Amelie 2022 (2) | Minor concerns  The authors employed a convenience sampling method, which could introduce potential selection bias. Nonetheless, the researchers determined that data saturation was achieved. As such, the number of participants was deemed adequate to yield comprehensive insights into the study's focus. | Minor concerns  Self-reported data may carry a potential for social desirability bias and could affect the credibility of the study findings. However, the study employed a semi-structured interview approach to counteract this effect. Furthermore, the researchers utilized open-ended questions and probes during the interviews to foster an environment that encouraged participants to share their genuine opinions. | No concerns  Data saturation was reached and in-depth data were reported, sufficient enough to support the study findings. | No concerns  The study findings are highly relevant to address the review’s objective. The study's results emphasize the necessity of decision aid tools for healthcare professionals and patients. Additionally, they underscore the significance of delivering precise and equitable information customized to the individual needs and preferences of women. | High confidence  Only minor concerns on the methodological limitation and coherence were observed. |
| Baptista 2020 (3) | Moderate concerns  Social desirability bias could have occur since study participants were aware that the interviewers were clinicians. However, the study involved rigorous data collection and analysis techniques during the study conducted. On another note, data analysis were done by two independent researchers guided by a theoretical framework during the conduct of thematic analysis. Also, the authors mentioned that the sample size was sufficient to gather comprehensive data. | Minor concerns  Since the researchers strongly believe that decision aids are helpful, this may have introduced information bias on how they analyze the data. However, this was minimized by constant comparison and discussion of the themes among authors. | No concerns  The data available were sufficient and relevant enough to support the study findings. | No concerns  The study findings are highly relevant to the review’s research objective. The population and the intervention included were also in line with the scope of the review. | Moderate confidence  Moderate concerns were observed relevant to the methodological limitations while minor concerns were observed in the coherence of this study. This is highly due to the awareness of participants to that the interviewers were clinicians and on the researcher’s bias perspective on the decision aids, which could strongly introduce social desirability bias and information bias, respectively. |
| Croes 2020 (4) | Minor concerns  The recruitment of experts was done through professional organizations, which could have introduced a selection bias and restricted the range of participants included. The use of Delphi method, however, ensured objective assessment of core elements of SDM for breast cancer screening as valued by patients and primary care providers. | No concerns  The addressed their research question and found nine core elements of SDM for women considering breast cancer screening, which were based on rigorous methodology and consensus among experts in the field. For this, the authors used a modified Delphi survey, widely accepted method for identifying and prioritizing consensus among experts in a particular field | Moderate concerns  The study did not include the perspectives of women at risk for breast cancer, leading to a gap in their representation in the study. Since structured quotations were also used, unique elements of SDM valued by patients and PCPs may not be captured. | Moderate concerns  No sufficient information of the women’s characteristics in order to determine whether they belonged to a vulnerable population.  The healthcare population, intervention, and settings are all highly relevant to address the review’s objectives. | Moderate confidence  Moderate concerns on the adequacy of the data available as well as the relevance of the study to the review’s research question were observed. Minor concerns relevant to the methodological limitations were also found. |
| Crothers 2016 (5)  (Mixed-method study) | No concerns  The authors reported that sample size was sufficient to reach data saturation. Data analysis were also conducted by multiple researchers and performed iterative round using immersion-crystallization techniques to come to agreement on key themes. Over, the methodological limitations were minimized using rigorous and reliable data collection and analysis approaches. | Minor concerns  Findings that captured patients’ attitudes and preferences about lung cancer screening and decision aid were clearly reported. However, the focus group discussion may introduce social desirability bias and group dynamics may have silenced dissenters. To reduce this bias, Facilitators sought input from all participants during the discussions. | No concerns  Data available were rich, detailed and in-depth enough to provide strong support of the identified themes. | Moderate concerns  The study findings were not mainly relevant to the review’s object with regards to patients or clinicians interest in terms of specific tool characteristics. However, some substantial quotations were captured relevant to the vulnerable population’s preferences in terms of SDM tool content, format and delivery strategies. | High confidence  Moderate and minor concerns regarding to the study’s relevance to the review’s objective, and the coherence of the study findings were observed. However, data available were rich and sufficient enough, and population included in the study provided substantial evidence on the preferences of vulnerable people in terms of the specific characteristics of the decision aids. |
| DuBenske 2021 (6)  (Mixed-method study) | Very minor concerns  Although the data collected during the interviews were rich and provided insights into participants’ experiences and perceptions of the SDM tool, there was no sufficient information whether the sample size was sufficient to reach data saturation. On the other hand, the data analysis was rigorously conducted using iterative process of content analysis and involvement of multiple experts to enhance the credibility of the findings. | Minor concerns  The potential selection, social desirability and provider bias might affect the credibility of the findings. However, authors reduced this bias by developing a well-structured questionnaire for both physician and patient survey. | Minor concerns  Data reported in the article were very limited and may missed to present unique preferences of patients and physicians. However, the amount of data presented to support main findings were adequate. | Moderate concerns  The study does not seem to be very relevant to the research question of the review. As the study mainly focuses decision aid satisfaction, limited preferences of the individuals regarding the specific characteristics of the SDM tool were only reported. | Moderate confidence  Minor concerns in methodological limitations, coherence and adequacy were found. Moderate concern, however, was observed in the relevance of the study findings to the review’s objective due to the limited data available regarding patients and physicians preference regarding the specific characteristics of decision aids. |
| Engelen 2017 (7) | Moderate concerns  There are several biases regarding the study participants as they performed a convenient sampling strategy. In addition, they included participants who were already interested in decision aid for early detection of cancer | Minor concerns  The credibility of their findings is questionable due to the potential following biases: selection bias and social desirability bias | Minor concerns  The thematic analysis from the study provides rich data regarding the potential facilitators and barriers of SDM tools for prostate cancer screening. To support their conclusions, quotations are provided. Data saturation is not specified. No contradictions or inconsistencies were found. | No concerns  The study outcomes are relevant and detailed in terms of facilitators and barriers of SDM tools for prostate cancer screening. | Moderate confidence  Even though the study findings answer the research question of the review, moderate concerns were found in the methodological limitations since the authors performed a study in a very specific population group, already interested in SDM, which could lead to biased results. Minor concerns were found in coherence and adequacy |
| Friedman 2012 (8) | Minor concerns  The sampling was purposive and convenience which might lead to selection bias.  Data was collected by a single interviewer which might introduced bias in the data collection process. | Moderate concerns  The credibility of their findings is questionable because it could have been social desirability, selection, and recall bias. In addition, as the focus group were guided by the researchers, there is a potential of researcher bias. Nevertheless, data source triangulation was used to mitigate these biases. | Minor concerns  The study did not differentiate between the experiences and perspectives of the men and women included. It is unclear if the quantity of the data is enough because it is not clear if data saturation was achieved in all the focus groups. | No concerns  The study addresses the research question of this review. They focused on vulnerable population, men and women, which provides rich information for the development of future SDM tools for prostate screening. | Moderate confidence  Only minor concerns were found in terms of methodological limitations, coherence and adequacy. |
| Halley 2014 (9) | Moderate concern  In the qualitative analysis, they do not specify if data saturation was reached, and it is not clear if data triangulation was performed.  Even though the sampling was random, the participants were mainly highly educated with high income and insured which indicates the possibility of selection bias | Minor concerns  The credibility of the findings in this study is questionable due to the evidence of selection bias and potential social desirability bias. | No concerns  The study used a mixed-methods approach to assess the effectiveness of one SDM tool vs another in three different medical conditions. In addition, it provides with quotations about patient’s perspectives on the decision support intervention | Moderate concerns  The study findings are relevant to answer the research question of the review. However, most of the participants had only one criterion for vulnerable population. | Moderate confidence  Moderate concerns were found in the methodological limitations and relevance. Minor concerns were found in the coherence aspect.. |
| Kuss 2021 (10) | Minor concerns  Small sample size of the physicians (only 9 were interviewed). | Moderate concerns  Credibility of their findings is affected by social desirability bias, and it is not clear if they performed data source triangulation. | Minor concerns  The quantity of the data might not be sufficient due to the small sample size, and it is not clear whether data saturation was reached. | Major concern  Because the participants were mostly highly educated data from these is not relevant to the review. However, data regarding physician perspectives is considered significant. | Low confidence  Major concerns were found in relevance and moderate concerns were found in adequacy of the data. Minor concerns were found in terms of methodological limitations and adequacy |
| Hernandez-Leal 2022 (11) | Minor concerns  Potential selection bias is observed due to the use of convenient sampling. However, such bias was minimized with the use of Delphi process, which ensures that the perspectives of all participants are taken into account and were based on the best available evidence. The study also employed rigorous methods using a mixed-method study design. | Minor concerns  Although potential social desirability and confirmation bias can be observed, which could reduce the credibility of the study findings, however, the authors minimized this bias by data source triangulation and by involving diverse panel of experts. | No concerns  The data supporting the study findings were adequate. No major inconsistencies or contradictions were observed. | No concerns  The population, settings and intervention of interest were all relevant to the review. | High confidence  only minors concerns on the methodological limitation and coherence were observed. |
| Maschke 2020 (12) | Minor concerns  Although theoretical saturation was achieved within the 23 patients and 17 PCPs being interviewed, imbalanced participation by race and exposure to SDM tool was observed. Nonetheless, data collection method was appropriate for the research question and were clearly described in the methods section. Finally, constant comparison technique was used to refine final themes. | No concerns  Reasons for such judgement:  The study findings address the key issues relevant to the lived experiences of mammography counseling among patients and PCPs. It also presented credible findings by providing supporting evidence using relevant quotations. | No concerns  Reasons for such judgement:  There is sufficient data to support the study findings and the data in-depth enough to provide a comprehensive understanding of the lived experiences of the patients who have limited health literacy. | No concerns  The population, settings and intervention of interest were all relevant to the review. | High confidence  only a minor concern on the diversity of population included in the study was observed. |
| Pannebakker 2019 (13) | Moderate concern  No sufficient information whether the sample size was appropriate to achieve data saturation. Nonetheless, they minimized this bias by conducting iterative process to sort the themes within the five main consolidated framework for implementation research(CFIR) domains. | Moderate concern  Although iterative process was done generate themes, only one researcher conduct the coding, which could lead to potential confirmation and information bias in the findings. Nonetheless, regular group meetings with the team was done to group data into higher-level analytical themes. | Minor concerns  The authors identified the universal lack of awareness of the use of the SDM tool (melanoma eCDS) during their consultation. Thus, there was no in-depth data sufficient to conduct further thematic analysis. There is also a limited quantity of data presented. However, rich verbatims were presented in the paper to support the findings. | No concerns  All domains, including the population, intervention and setting are highly relevant in the review. | Moderate confidence  There are moderate concerns observed in the methodology and coherence of the study. |
| Reese 2022 (14) | Minor concerns  Small sample size was observed without any mention of data saturation. However, the authors conducted a thorough needs assessment and used multiple data source to gather information from primary care providers. They also used a multidisciplinary team to analyze the data and develop the implementation strategy. | No concerns  The study findings presented key issues that are highly relevant to the research question. They also presented range of quotations to support the identified theme and provided specific examples of the issues identified in the study. Moreover, the authors provided a rigorous description of their data collection and analysis approach, which enhances the transparency and trustworthiness of the study findings. | Minor concerns  Although the author did not mentioned about achieving data saturation, they were able to present a rich and diverse range of quotations on the needs and perspectives of the primary care providers. | Serious concerns  The intervention and context of interest is not directly relevant in the review. The review specifically focused on SDM tools, but the study only partially addressed this topic. While the study did mention a decision support tool as part of the implementation strategy, the study’s research question was different from the review’s topic of interest. | Low confidence  A serious concern on the relevance of the study to the review’s scope is observed. Minor concerns were also observed in the methodological limitation and adequacy of data to support study findings. |
| Schapira 2016 (15) | Minor concerns  The study was conducted at a single academic medical center, which may limit the generalizability of the findings to other settings or populations. They also used convenience sampling, which may not represent broader population eligible for screening and might have attracted more patients who were more interested or motivated to participate in the screening process. | No concerns  Study findings addressed the key issues relevant to the research questions. Moreover, triangulating quantitative findings with the qualitative surveys and use of multiple methods in data collection and analysis were done to enhance the validity and reliability of findings. It also addressed important gaps in the literature related to patient preferences and decision-making in the context of cancer screening | Minor concerns  There was no sufficient evidence to determine whether data was enough to reach data saturation. However, rigorous approach during the thematic analysis was performed and diverse range of participants were included. Moreover, the authors presented rich verbatims to support every identified theme. | Moderate concerns  The context did not mainly focused on the preferences of the patients in terms of SDM tool characteristics. However, they provided information about the importance of providing clear, accurate information to patients about the potential benefits and harms of screening, and on addressing patient’s concern and uncertainties. Patient preferences and decision-making in cancer screening was also addressed in this study. | Moderate confidence  There are minor and moderate concerns observed in the methodological limitations and relevance of the study to the review’s topic and intervention of interest, respectively. |
| Schonberg 2019 (16) | Moderate concerns  The study did not involve patient participation, limiting its generalizability, despite the decision aids being intended for older women. However, data collection was rigorously conducted by trained qualitative researchers, and data analysis was performed by at least two independent researchers using an iterative process. The team also had regular meetings to ensure transparent and reliable findings. | No concerns  The study findings addressed the research question very well and were supported with rich verbatims. The findings were also presented in a detailed and comprehensive way that the readers can follow. No inconsistencies or contradictions in the data or findings were observed. | Minor concerns  There is limited diversity among participants, who were more white and female primary care staff. However, the data collected was still sufficient and of good quality, supported by trained researchers and well-presented quotations on ideas for implementing decision aids for cancer screening. | No concerns  The population, intervention and settings are all highly relevant to the review’s qualitative research objective. | Moderate confidence  There are only minor concerns observed in the methodological limitations and adequacy of findings due to limited diversity of participants involved. |
| Schwartz 2021 (17) | Very minor concerns  The analysis was done by multiple researchers. However, there is limited information whether sample size was sufficient to achieve data saturation. Nonetheless, the researchers did a thorough analysis of the data and identified key themes and patterns that emerged from the discussions. This may suggest that they have reached a point of data saturation. | No concerns  The findings were adequate, credible and relevant enough to address the research question. The findings provided valuable insights into layperson views about decision aids. | No concerns  The data was rich and in-depth enough to support the study findings. The authors used a public deliberation process related to decisions aids to gather data from a diverse groups of participants. Moreover, they presented the findings in a clear and concise manner, using quotations from the participants to illustrate key points. | No concerns  The population, intervention, context, and setting are all relevant to address the review’s qualitative objective. | High confidence  Only a very minor concern related to methodological limitation was observed. |
| Tatari 2020 (18) | Very minor concerns  Since minorities were involved, social desirability bias might be present. The authors minimized this bias by involving trained interviewed to conduct the interviews and ensured that participants felt comfortable sharing their honest opinions and experiences. Moreover, the researchers conducted groups until they felt that no new information was emerging. Diverse range of participants were also included in the study and team approach was used in the data analysis. | No concerns  The study findings strongly addressed the research question relevant to the preferences of ethnic minority women regarding tailored SDM intervention. | No concerns  Rich verbatims and detailed quotations were presented to support the study findings. | No concerns  The population, intervention, context, and settings are highly relevant to address the review’s qualitative objective. The study mainly focused on ethnic minority. Preferences in terms of SDM tool characteristics were well-reported. | High confidence  A very minor concern was only observed on the methodological limitation. |
| Toledo-Chavarri 2016 (19) | Very minor concerns  The study only conducted two focus group discussions of professionals with heterogenous profile, which presents too diverse perspectives. The study achieved data saturation and addressed potential social desirability bias by involving trained researchers and ensuring participant anonymity. A team of researchers also conducted the data analysis in a systematic and rigorous manner, following a pre-determined protocol. | No concerns  Study findings were coherent with the research question of the study. Credibility of the study findings were also observed as sufficient supporting quotations were provided in a very detailed manner. | No concerns  Data were adequate and credible enough to support the findings of the study. Moreover, rich quotations were presented in a comprehensive and clear manner. No inconsistencies were observed in the presented quotations in relation to the study findings. | No concerns  The study population, intervention, context and settings were highly relevant to address the review’s qualitative research question. | High confidence  Only a very minor concern was observed regarding methodological limitations of the study. |
| Vahabi 2011 (20) | Minor concerns  While there was no clear indication of whether the sample size was sufficient to reach data saturation, the study collected rich and in-depth data that supported the study’s findings. The study was also conducted in English, which may limit the participation of Iranian immigrant women who do not speak English. The data analysis process was also conducted rigorously by two independent researchers, and discrepancies were resolved through discussion an consensus. | No concerns  Study findings directly addressed the research question and credibility of the study findings were observed as study conduct was well-designed. The study was also novel in that it provided insights into the breast cancer and screening information needs and preferred communication medium of Iranian immigrant women. | Minor concerns  There was no clear indication whether the amount of data available was sufficient enough to support the study findings. However, quality or richness as well as consistencies of the data were observed. | No concerns  The study population, intervention, context and settings were highly relevant to address the review’s qualitative research question. | High confidence  Few minor concerns relevant to the methodological limitations and adequacy of data collected were observed. |
| Wiener 2018 (21) | Minor concerns  Selection bias could be observed since study participants were recruited from early adopter of lung cancer screening programs. Nonetheless, authors minimized this bias by including diverse range of ages, races, and educational level. On another note, clinicians included in this study were all involved in the lung cancer screening program and had experience with SDM. | No concerns  The study findings are credible, as the authors used multiple strategies to ensure the trustworthiness and rigor of the data analysis. This includes the use of a team-based approach to coding and analyzing the data, and conduct of member checking with study participants to ensure the accuracy of findings. | Very minor concerns  The generalizability of the study findings may be limited to specific context of the study population. However, the authors presented numerous quotations from the interviews to illustrate key themes and concepts. In-depth and detailed exploration of their perspectives on SDM was also observed. | Moderate concerns  Patient participants was excluded in the review as no sufficient information was provided to identify them as predominantly vulnerable. Thus, only clinician participants were the only relevant population to address the review’s qualitative research question. | Moderate confidence  The lack of sufficient detail regarding the patient characteristics has made it difficult to assess the extent to which the study findings can be extrapolated to other patient groups. |

**References**

1. Akanuwe JNA, Black S, Owen S, Siriwardena AN. Communicating cancer risk in the primary care consultation when using a cancer risk assessment tool: Qualitative study with service users and practitioners. Health Expectations. 2020 Apr 1;23(2):509–18.

2. Amélie AE, Ruelle Y, Frèche B, Houllemare M, Bonillo A, Bouaziz L, et al. What do women and healthcare professionals expect of decision aids for breast cancer screening? A qualitative study in France. BMJ Open. 2022 Mar 15;12(3).

3. Baptista S, Heleno B, Pinto M, Guimarães B, China D, Ramos JP, et al. Translation and cultural adaptation of a prostate cancer screening decision aid: A qualitative study in Portugal. BMJ Open. 2020 Mar 25;10(3).

4. Croes KD, Jones NR, DuBenske LL, Schrager SB, Mahoney JE, Little TA, et al. Core Elements of Shared Decision-making for Women Considering Breast Cancer Screening: Results of a Modified Delphi Survey. J Gen Intern Med. 2020 Jun 1;35(6):1668–77.

5. Crothers K, Kross EK, Reisch LM, Shahrir S, Slatore C, Zeliadt SB, et al. Patients’ attitudes regarding lung cancer screening and decision aids: A survey and focus group study. Vol. 13, Annals of the American Thoracic Society. American Thoracic Society; 2016. p. 1992–2001.

6. DuBenske L, Ovsepyan V, Little T, Schrager S, Burnside E. Preliminary Evaluation of a Breast Cancer Screening Shared Decision-Making Aid Utilized Within the Primary Care Clinical Encounter. J Patient Exp. 2021;8.

7. Engelen A, Vanderhaegen J, Van Poppel H, Van Audenhove C. The use of decision aids on early detection of prostate cancer: views of men and general practitioners. Health Expectations. 2017 Apr 1;20(2):221–31.

8. Friedman DB, Thomas TL, Owens OL, Hébert JR. It Takes Two to Talk About Prostate Cancer: A Qualitative Assessment of African American Men’s and Women’s Cancer Communication Practices and Recommendations. Am J Mens Health. 2012 Nov;6(6):472–84.

9. Halley MC, Rendle KAS, Gillespie KA, Stanley KM, Frosch DL. An exploratory mixed-methods crossover study comparing DVD- vs. Web-based patient decision support in three conditions: The importance of patient perspectives. Health Expectations. 2015 Dec 1;18(6):2880–91.

10. Kuss K, Adarkwah CC, Becker M, Donner-Banzhoff N, Schloessler K. Delivering the unexpected—Information needs for PSA screening from Men’s perspective: A qualitative study. Health Expectations. 2021 Aug 1;24(4):1403–12.

11. Hernández-Leal MJ, Codern-Bové N, Pérez-Lacasta MJ, Cardona A, Vidal-Lancis C, Carles-Lavila M. Development of support material for health professionals who are implementing Shared Decision-making in breast cancer screening: Validation using the Delphi technique. BMJ Open. 2022 Feb 1;12(2).

12. Maschke A, Paasche-Orlow MK, Kressin NR, Schonberg MA, Battaglia TA, Gunn CM. Discussions of Potential Mammography Benefits and Harms among Patients with Limited Health Literacy and Providers: “Oh, There are Harms?” J Health Commun. 2020;25(12):951–61.

13. Pannebakker MM, Mills K, Johnson M, Emery JD, Walter FM. Understanding implementation and usefulness of electronic clinical decision support (eCDS) for melanoma in English primary care: A qualitative investigation. BJGP Open. 2019 Apr 1;3(1).

14. Reese TJ, Schlechter CR, Kramer H, Kukhareva P, Weir CR, Del Fiol G, et al. Implementing lung cancer screening in primary care: Needs assessment and implementation strategy design. Transl Behav Med. 2022 Feb 1;12(2):187–97.

15. Schapira MM, Aggarwal C, Akers S, Aysola J, Imbert D, Langer C, et al. How patients view lung cancer screening: The role of uncertainty in medical decision making. Ann Am Thorac Soc. 2016 Nov 1;13(11):1969–76.

16. Schonberg MA, Jacobson AR, Aliberti GM, Hayes M, Hackman A, Karamourtopolous M, et al. Primary Care–Based Staff Ideas for Implementing a Mammography Decision Aid for Women 75+: a Qualitative Study. J Gen Intern Med. 2019 Nov 1;34(11):2414–20.

17. Schwartz PH, O’Doherty KC, Bentley C, Schmidt KK, Burgess MM. Layperson Views about the Design and Evaluation of Decision Aids: A Public Deliberation. Medical Decision Making. 2021 Jul 1;41(5):527–39.

18. Tatari CR, Andersen B, Brogaard T, Badre-Esfahani S, Jaafar N, Kirkegaard P. The SWIM study: Ethnic minority women’s ideas and preferences for a tailored intervention to promote national cancer screening programmes—A qualitative interview study. Health Expectations. 2021 Oct 1;24(5):1692–700.

19. Toledo-Chávarri A, Rué M, Codern N, Carles-Lavila M, Perestelo L, Pérez-Lacasta MJ, et al. A qualitative study on a decision aid for breast cancer screening: Views from women and health professionals. Eur J Cancer Care (Engl). 2017 May 1;26(3).

20. Vahabi M. Breast cancer and screening information needs and preferred communication medium among Iranian immigrant women in Toronto. Health Soc Care Community. 2011 Nov;19(6):626–35.

21. Wiener RS, Koppelman E, Bolton R, Lasser KE, Borrelli B, Au DH, et al. Patient and Clinician Perspectives on Shared Decision-making in Early Adopting Lung Cancer Screening Programs: a Qualitative Study. J Gen Intern Med. 2018 Jul 1;33(7):1035–42.
