# Supplementary material for: Mixed-Method Systematic Review and Meta-Analysis of Shared Decision-Making Tools for Cancer Screening
Source: Cancers (Basel). 2023 Jul 29;15(15):3867. doi: 10.3390/cancers15153867 (PMC10417450; doi:10.3390/cancers15153867)
Supplement: Supplementary file 1 [file cancers-15-03867-s001.zip › cancers-2455744-supplementary/Supplementary file_6_reasons for exclusion copy.docx]

**File S6**. **Characteristics of excluded studies** [ordered by number]

**Table S4.** Reasons for exclusion of each study.

| **Study ID** | | **Reason for excluding** | | **Tagged reason** | |  |
| --- | --- | --- | --- | --- | --- | --- |
| Aijaz 2021 | | Focuses on the barriers to lung cancer screening instead of SDM tool preferences in terms of content, format and/or delivery strategies | | Wrong context | |  |
| Akbari 2020 | | This study did not include outcomes relevant to the SDM process or on the cognitive-affective outcomes of individuals considering cancer screening. | | Wrong outcome | |  |
| Allen 2021 | | The study is a pilot-testing of a decision aid with only 43 study participants. | | Wrong study design | |  |
| Amornsiripanitch 2021 | | Did not evaluate the effectiveness of an SDM tool for cancer screening but rather focuses on the influence of demographic factors on women’s perceptions and preferences regarding cancer breast risk communication. | | Wrong context | |  |
| Almyroudi 2011 | | This employed a cross-sectional study design, which makes it hard to determine the effect of an SDM tool on SDM process and cognitive-affective outcomes of an individual. | | Wrong study design | |  |
| Aubin-Auger 2013 | | This qualitative study did not involve any vulnerable populations. | | Wrong population | |  |
| Banerji 2017 | | The full text of this study could not be retrieved. | | Not retrievable | |  |
| Baptista 2020 | | This is a study protocol. | | Wrong study design | |  |
| Bass 2013 | | This is a development study of a computer touch-screen interactive colorectal screening decision aid and not an effectiveness study. | | Wrong study design | |  |
| Bellinger 2019 | | No full-text available online, but only abstract. | | No full-text available | |  |
| Bone 2013 | | This is a protocol. | | This is a protocol. | |  |
| Bowen 2011 | | Data used in this study were based only on national surveys, which makes it hard to determine the specific SDM tool used by the participants. | | Wrong study design | |  |
| Brackett 2010 | | There is no well-defined SDM tool intervention reported in both the study and results were presented in an aggregated-level. | | Wrong study design | |  |
| Brackett 2015 | | This study did not present any relevant outcomes about SDM process nor individual-level outcomes | | Wrong outcome | |  |
| Braillon 2015 | | This is a review/editorial. No actual involvement of participants. | | Wrong study design | |  |
| Brenner 2014 | | This study did not evaluate any SDM tool for cancer screening but rather on the different value clarification approaches for colorectal cancer screening decision-making. | | Wrong intervention | |  |
| Brenner 2016 | | This is a development study that only focuses on practical model for patient decision support but did not evaluate the effectiveness of the SDM tool. | | Wrong study design | |  |
| Brenner 2018 | | No SDM tool is involved in this study. | | Wrong intervention | |  |
| Brinker 2017 | | Listed only as poster presentation, no full-text retrievable. | | Not retrievable | |  |
| Brittain 2012 | | No SDM tool presented in this study and only presented findings on the factors associated with informed decision-making. | | Wrong context | |  |
| Brown 2019 | | This qualitative study did not involve any SDM tool topics for cancer screening. | | Wrong context | |  |
| Burnside 2022 | | Data on the individual baseline information of the participants were not presented and the design is not ideal to present evidence on the effectiveness of a SDM tool | | Wrong study design | |  |
| Buyan 2017 | | This study did not evaluate a different intervention for cancer screening and not an SDM tool. | | Wrong intervention | |  |
| Cadet 2021 | | The SDM tool is not well-defined in the report, and we have no idea whether a specific tool was used or not. | | Wrong intervention | |  |
| Carter-Harris 2020 | | The study is a pilot study with only 60 study participants. | | Wrong study design | |  |
| Caverly 2021 | | This study focuses only on the SDM intervention in general and not on the effects on the use of a shared decision-making tool. | | Wrong context | |  |
| Christy 2013 | | This study did not include any relevant outcomes of the SDM process but only on associated factors influencing screening behaviors. | | Wrong context | |  |
| Clark 2022 | | Did not evaluate any shared decision-making tool but only on a specific additional information about incidental findings | | Wrong context | |  |
| Costanza 2011 | | The study is a pilot study of telephone counseling. | | Wrong study design | |  |
| Cunich 2011 | | This is a pilot-testing study design of a web-based decision analytic tool and not an effectiveness study. | | Wrong study design | |  |
| Davis 2011 | | This qualitative study did not include relevant outcomes on preferred SDM tool content, format, or delivery strategies. | | Wrong outcome | |  |
| Denizard-Thompson 2020 | | This study focuses on barriers and facilitators of an SDM intervention and not on the preferences of SDM tool content, format, and delivery strategies. | | Wrong context | |  |
| Dharod 2019 | | This study did not include any relevant outcomes on the SDM process or on the cognitive-affective outcomes of the participants. | | Wrong outcome | |  |
| Dickinson 2019 | | This study focuses only on the critical factors that affect the SDM process in general. | | Wrong intervention | |  |
| Dilliard 2010 | | This study uses a cross-sectional study design, which is not sufficient to evaluate the effectiveness of an SDM tool on SDM process and cognitive-affective outcome. | | Wrong study design | |  |
| Dobbins 2020 | | This study is still on-going | | On-going study | |  |
| Dodd 2019 | | This study did not involve any SDM tool intervention. | | Wrong intervention | |  |
| Dolan 2002 | | This study was published before 2010. | | Published before 2010 | |  |
| Dolan 2014 | | This study did not involve any SDM tool for cancer screening. | | Wrong context | |  |
| Dolan 2015 | | This study is more focused on communication strategic intervention rather than on the use of a shared decision-making tool for cancer screening. | | Wrong intervention | |  |
| DuBenske 2017 | | The outcomes captured in this study were only relevant to barriers and facilitators of SDM intervention and not on the participant’s preferred SDM tool content, formats, or delivery strategies. | | Wrong outcome | |  |
| Duren-Winfield 2015 | | This study did not include relevant outcomes on the SDM process and on the cognitive-affective outcomes of an individual. | | Wrong outcome | |  |
| Early 2014 | | This study only presents findings on patient attitudes in colon cancer screening and not on SDM tool’s preferences. | | Wrong context | |  |
| Eden 2020 | | Focuses on factors associated with the use of breast cancer risk assessment and patient decision aid, and not on its effects. | | Wrong study design | |  |
| Ekberg 2014 | | There is no well-defined SDM tool intervention presented in the study, and employs a cross-sectional study design. | | Wrong study design | |  |
| Eikin 2017 | | Involved participants who already had mammography screenings and the SDM tool is not mainly intended to support the SDM process. | | Wrong population and intervention | |  |
| Elliot 2022 | | This study involved multiple SDM tools meant to support SDM process to different cancer screening. As the tool is not well-defined for each cancer screening, specifically its content, we decided to exclude this study. | | Wrong intervention | |  |
| Elliott 2022 | | This study only included health institutional-level outcome measures. | | Wrong outcome | |  |
| Emery 2018 | | Pilot-testing and development study and did not include actual participants eligible for cancer screening. Moreover, the study did not present any outcome that is within the scope of our review | | Wrong study design | |  |
| Fagan 2020 | | This is a pilot testing and development study and does not present any effectiveness findings on a SDM tool. | | Wrong study design | |  |
| Felder 2022 | | This study is still on-going. | | On-going study | |  |
| Fraenkel 2016 | | The study focused solely on the factors associated with lung cancer screening decision rather than the effects of using a SDM tool for cancer screening. | | Wrong context | |  |
| Frencher 2016 | | The study is a non-randomized pilot study. | | Wrong study design | |  |
| Frosch 2007 | | This study was conducted and published before 2010. | | Year published before 2010 | |  |
| Frosch 2011 | | This study did not use any individual-level data but rather by practice. | | Wrong population | |  |
| Fukunaga 2022 | | This is a pilot study of an encounter decision aid for lung cancer screening and not an effectiveness study. | | Wrong study design | |  |
| Gabel 2018 | | This qualitative study did not include outcomes on preferred SDM tool content, format, or delivery strategies. | | Wrong outcome | |  |
| Galpin 2017 | | This study is not relevant to SDM tools for cancer screening but solely explores the motivation of stakeholders when screening. | | Wrong context | |  |
| Garcia-Alonso 2017 | | This study evaluated SDM intervention in general instead of an SDM tool. Moreover, there is no comparator or control group included for this study. | | Wrong intervention | |  |
| Gaster 2010 | | No primary data involved in this study and did not evaluate any SDM tool. | | Wrong context | |  |
| Gatting 2021 | | This study only involves content analyses review and did not involve actual study participants. | | Wrong study design | |  |
| Giguere 2012 | | This qualitative study did not involve vulnerable populations or clinicians. | | Wrong population | |  |
| Gourlay 2010 | | This study did not involve any SDM tool intervention and only described the primary care patients’ perception on SDM process for cancer screening, | | Wrong context | |  |
| Green 2015 | | This study included symptomatic patients. | | Wrong population | |  |
| Griffith 2008 | | This is a pilot randomized trial on the newly developed decision aid and is not an effectiveness study. Moreover, there is no clear definition as to whether the decision aids the SDM process. | | Wrong study design and intervention | |  |
| Griffin 2022 | | This is a study protocol. | | Wrong study design | |  |
| Gross 2015 | | There is no sufficient information on the baseline characteristics of the study participants to distinguish them into vulnerable populations. | | Wrong population | |  |
|  | |  | |  | |  |
| Gwende 2015 | | This study did not include vulnerable populations. | | Wrong population | |  |
| Gunn 2021 | | This study did not evaluate any SDM tool for cancer screening. | | Wrong context | |  |
| Gunn 2021 | | This qualitative study did not capture any SDM tool preferences of the vulnerable population or clinicians. | | Wrong outcome | |  |
| Han 2013 | | The study did not have any well-defined SDM tool intervention and used retrospective data to evaluate the tool’s effect. | | Wrong study design | |  |
| Han 2019 | | None of the outcomes measured is within the scope of our review and no information on the baseline characteristics of the included participants. | | Wrong context | |  |
| Hamdiui 2022 | | This study did not cover outcomes on the participant’s preferences in terms of SDM tool’s content, format or delivery strategies for the qualitative review. | | Wrong outcome | |  |
| Harper 2021 | | The study did not evaluate any SDM tool for cancer screening and outcomes were not relevant to SDM process nor cognitive-affective outcome of an individual. | | Wrong context | |  |
| Harris 2008 | | Did not focus on the effects of a shared decision-making tool but on the SDM intervention in general. | | Wrong intervention | |  |
| Harry 2020 | | Although both intervention arms were highly relevant for the review, this study only presented the pre-implementation barriers and facilitators at a system-level findings. | | Wrong context | |  |
| Harry 2022 | | This involves a survey [cross-sectional] study design, and the intervention in this study was not clearly defined. | | Wrong study design | |  |
| Hart 2016 | | This is a review on the development and evaluation of a lung cancer screening decision aid. | | Wrong study design | |  |
| Hawley 2015 | | This study did not include outcomes relevant to the SDM process or on the cognitive-affective outcomes of individuals considering cancer screening. | | Wrong outcome | |  |
| Henderson 2011 | | This qualitative study did not involve any SDM tool and outcomes were not relevant to address the qualitative review. | | Wrong context | |  |
| Hersch 2020 | | No available full-text report as this is only a poster presentation. | | Non-retrievable | |  |
| Hoffman 2010 | | No SDM tools being actively employed by the investigators to facilitate SDM. | | Wrong context | |  |
| Hoffman 2014 | | This study did not present any clear SDM tool description and the study design is retrospective in nature. | | Wrong study design | |  |
| Hoffman 2016 | | Results from this study is based on a national survey, thus, the study design is not sufficient to present reliable evidence on the effectiveness of a SDM tool. | | Wrong study design | |  |
| Hyams 2021 | | This study did not focus on the SDM tool but rather on the screening preferences. | | Wrong context | |  |
| Ilic 2008 | | This study was conducted and published before 2010. | | Year published before 2010 | |  |
| Ilic 2013 | | This qualitative study did not involve any SDM tools nor outcomes on the user’s preferences in terms of SDM tool content, format, or delivery strategies. | | Wrong context | |  |
| Imaeda 2010 | | No well-defined SDM tool used and no identified comparator. Moreover, outcomes in this study were outside the scope of our review. | | Wrong intervention | |  |
| Jimbo 2013 | | This qualitative study focuses only on perceived barriers and facilitators of an SDM tool and not on user preferences in terms of its content, format, and delivery strategies. | | Wrong context | |  |
| Kanodra 2016 | | The SDM tool in this study was not clearly defined. | | Wrong intervention | |  |
| Kaplan 2021 | | This study used a cross-sectional design, which is not sufficient to evaluate the effectiveness of a SDM tool on SDM process and cognitive-affective outcome of an individual. | | Wrong study design | |  |
| Kassan 2012 | | This qualitative study did not involve vulnerable populations or clinicians. | | Wrong population | |  |
| Katz 2012 | | This qualitative study did not include outcomes that are relevant to SDM tool preferences of vulnerable populations or clinicians. | | Wrong outcome | |  |
| Kim 2017 | | No SDM tool was described in the paper and outcomes were outside the scope of our review. | | Wrong context | |  |
| Kim 2018 | | This study is mainly focused on risk communication and predictors of appropriate screening and not on SDM tool’s effect on SDM process or on the cognitive-affective outcomes of the participants. | | Wrong context | |  |
| Klein 2016 | | This study is relevant to address secondary objectives of the review, but study participants are not dominantly vulnerable populations. | | Wrong population | |  |
| Koo 2017 | | The study did not evaluate any SDM tool that supports the SDM process. | | Wrong context | |  |
| Krist 2020 | | There is no well-defined description of an SDM tool presented in this study. | | Wrong intervention | |  |
| Kristler 2018 | | The study did not include relevant outcomes on the SDM process or on the cognitive-affective outcomes of an individual. | | Wrong outcome | |  |
| Krist 2007 | | This study was published before 2010. | | Published before 2010 | |  |
| Krist 2017 | | This is a pilot testing and development study of a personalized educational material and is not an effectiveness study. | | Wrong study design | |  |
| Kukaftka 2015 | | Focuses only on barriers for decision aid utilization and not on the preferences of vulnerable populations in terms of SDM tool content, format, and delivery strategies. | | Wrong context | |  |
| Kushalnagar 2020 | | No well-defined SDM tool and the study design is retrospective in nature. | | Wrong study design | |  |
| Lafata 2014 | | This study did not include any relevant outcomes on the SDM process or on the cognitive-affective outcomes of an individual. | | Wrong outcome | |  |
| Leone 2013 | | No sufficient information about the baseline characteristics of the participants and could not identify whether the population is vulnerable or not. | | Wrong population | |  |
| Leyva 2016 | | This study did not present a well-defined SDM tool that was being evaluated and data were collected from a national survey. | | Wrong study design | |  |
| Lowrey 2022 | | Results were presented by cluster and no individual level data were reported. | | Wrong outcome | |  |
| Li 2013 | | Used a retrospective survey study design, which is not sufficient to measure effects of a SDM tool intervention. | | Wrong study design | |  |
| Linder 2014 | | This study only focuses on physical behavior rather to promote informed decision and not on the effectiveness of an SDM tool. | | Wrong context | |  |
| Liu 2018 | | This study only focuses on risk score based educational tools and did not evaluate the effectiveness of the tool. | | Wrong context | |  |
| Lillie 2017 | | No well-defined SDM tool and outcomes were focused on associated factors and not on the effects of an intervention. | | Wrong context | |  |
| Makoul 2009 | | Multimedia patient education programs were not described as an SDM tool that could support informed decision-making in cancer screening. | | Wrong intervention | |  |
| Maleyeff 2020 | | This study is more relevant to modeling study rather than to intervention study. | | Wrong context | |  |
| Mambourg 2018 | | This study is not presented in English, but rather in French. | | Wrong language | |  |
| Matthias 2020 | | This study only focuses on the barriers and facilitators of a risk prediction tool. | | Wrong context | |  |
| Mazzone 2017 | | There is no sufficient information on the baseline characteristics of study participants to distinguish vulnerable populations. | | Wrong population | |  |
| Mbah 2015 | | This is a protocol. | | This is a protocol. | |  |
| Miles 2013 | | No SDM tool presented in the study. | | Wrong intervention | |  |
| Miller 2012 | | Intervention included in this study is not for cancer screening. | | Wrong context | |  |
| Miller 2017 | | This study did not present any relevant outcome on the SDM tool preferences of vulnerable populations or clinicians, in terms of content, format and delivery strategies. | | Wrong outcome | |  |
| Misra-Hebert 2018 | | The outcomes measured in this study were not relevant to the SDM process or on the cognitive-affective outcomes of the participants. | | Wrong outcomes | |  |
| Molokwu 2017 | | This study did not evaluate any SDM tool but rather on the decision-making preferences in colorectal cancer screening program. | | Wrong context | |  |
| Mota 2019 | | The intervention was not intended to support SDM process and outcome measured was not relevant to SDM process nor cognitive-affective outcome of the participants. | | Wrong context | |  |
| Nadler 2022 | | This study focuses only on the determinants, including facilitators and barriers of breast cancer screening. | | Wrong context | |  |
| Neil 2022 | | This study did not present a well-defined SDM tool for cancer screening and there is no sufficient information to conclude that an SDM tool intervention was evaluated. | | Wrong intervention | |  |
| Nishi 2021 | | No well-defined description on the decision aid and outcome measures were only retrospectively assessed. | | Wrong study design | |  |
| Nowakowski 2012 | | This is a commentary and does not involve actual participants. | | A commentary paper | |  |
| O’Farrell 2012 | | This study did not provide sufficient information whether or not an SDM tool for cancer screening was evaluated. | | Wrong intervention | |  |
| Owens 2019 | | This study only investigated associated factors of a decision aid. | | Wrong study design | |  |
| Owens 2022 | | This is a pilot and development study design and is not an effectiveness study of an SDM tool. | | Wrong study design | |  |
| Ozzane 2010 | | There is no actual involvement of participants in this study nor any SDM tool interventions. | | Wrong context | |  |
| Parekh 2021 | | This study did not assess the effects of any SDM tool intervention but rather on the barriers and facilitators to cancer screening only. | | Wrong context | |  |
| Percac-Lima 2018 | | The patient navigation for lung cancer screening were more focused on decision-coaching and training rather than the effect of a shared decision-making tool for cancer screening | | Wrong intervention | |  |
| Petrova 2015 | | The outcome measures in this study were not focused on the SDM process nor on the cognitive-affective outcomes of the people facing cancer screening. | | Wrong outcome | |  |
| Pignone 2011 | | This study did not include an SDM tool but rather decision aids as an awareness tool about cancer screening. | | Wrong intervention | |  |
| Pignone 2013 | | This study did not evaluate any SDM tool for cancer screening. | | Wrong intervention | |  |
| Politi 2020 | | This study on BREASTChoice tool is not for cancer screening but for patients that have undergone mastectomy for reconstruction. | | Wrong context | |  |
| Price-Haywood 2017 | | No reports available, including in ClinicalTrial.org website. Moreover, description of the intervention is not relevant to the scope of this review. | | Wrong intervention, no report available | |  |
| Prochaska 2016 | | The study design is not appropriate to evaluate the effectiveness of a patient decision aid. | | Wrong study design | |  |
| Puccinelli-Ortega 2022 | | This qualitative study is solely focused on the barriers and facilitators of a decision-making tool and not on SDM tool preferences. | | Wrong context | |  |
| Raz 2021 | | This study did not include an SDM tool intervention that is intended to support the SDM process. | | Wrong intervention | |  |
| Reuland 2017 | | Unable to ascertain whether the tool’s intent was to support shared decision-making process or to increase screening rates | | Wrong intervention | |  |
| Richter 2021 | | Focuses on screening test preferences and not on their preferences in terms of SDM tool content, format and/or delivery strategies | | Wrong outcome measures | |  |
| Rim 2019 | | No actual involvement of participants in this study and outcomes were not relevant to the SDM process nor the cognitive-affective outcomes of the participants. | | Wrong context | |  |
| Rupert 2013 | | This is a pilot testing of an interactive decision support tool and does not measure the effectiveness of the tool. | | Wrong study design | |  |
| Ruzek 2016 | | The tool was not intended to support SDM process but rather on motivation to screen. | | Wrong intervention | |  |
| Sacoda 2020 | | Although the study aimed at supporting an SDM process, there is no tangible and clearly defined SDM tool being evaluated. | | Wrong intervention | |  |
| Salzman 2020 | | No full-text retrievable as only an abstract is presently published. | | Non-retrievable | |  |
| Saman 2019 | | This study did not include any relevant outcomes on the SDM process or on the cognitive-affective outcomes. Moreover, SDM tools were not well-defined, with no sufficient information on the tool’s intent to support the SDM process. | | Wrong outcome | |  |
| Saman 2021 | | Did not evaluate a shared decision making tool. | | Wrong context | |  |
| Samimi 2022 | | This is a commentary. | | Wrong study design | |  |
| Sanders 2016 | | This study only focuses on determining the associated factors on patient-reported discussion of screening with providers and did not evaluate the effectiveness of an SDM tool. | | Wrong context | |  |
| Sava 2018 | | This study did not have a well-defined SDM tool intervention and no clear comparator group. Moreover, it only involves secondary data analyses rather than using primary data. | | Wrong study design | |  |
| Sava 2022 | | This is a modeling study rather than an intervention study. | | Wrong context | |  |
| Scalia 2017 | | The study evaluated a tool that is not in the context of cancer screening and baseline characteristics of the study populations were not clearly presented. | | Wrong context | |  |
| Scalia 2018 | | This study uses a cross-sectional study design, which is not sufficient to evaluate the effectiveness of an SDM tool on SDM process and cognitive-affective outcome. | | Wrong study design | |  |
| Scalia 2019 | | This report did not involve actual participants as it only presents study documentations/lessons learned to improve usability of decision aids. | | Wrong context | |  |
| Scariati 2015 | | This study involves a pilot study design for a decision aid in screening mammography and not an actual effectiveness study. | | Wrong study design | |  |
| Scharger 2019 | | This is a commentary. | | Wrong study design | |  |
|  | |  | |  | |  |
| Schapira n.d. | | Unable to retrieve full text. | | Not retrievable | |  |
| Schapira 2022 | | This is a pilot-testing and development study of a web-based decision aid for lung cancer screening and not an effectiveness study. | | Wrong study design | |  |
| Schloessler 2014 | | This is a pilot testing and development study of a transactional decision aid. | | Wrong study design | |  |
| Schonberg 2014 | | This is a pilot and development study of a decision aid for mammography screening rather than an actual effectiveness study. | | Wrong study design | |  |
| Schroy 2014 | | This is a cross-sectional survey, and we consider this study design to be insufficient in assessing the effects of an SDM tool on the SDM process and the cognitive-affective outcome of participants. | | Wrong study design | |  |
| Schroy 2015 | | This study did not measure any relevant outcomes on the effect of a SDM tool on SDM process, cognitive-affective outcomes nor on other behavioral outcomes such as intention to screen, screening uptake or tests ordered. | | Wrong outcomes | |  |
| Schwartz 2017 | | Unable to ascertain if the tool supports shared decision-making process instead of motivation to take the stool test | | Wrong intervention | |  |
| Seaman 2018 | | This qualitative study did not include any vulnerable populations or clinicians. | | Wrong population | |  |
| Sferra 2017 | | Listed only as poster presentation, no full-text retrievable. | | Not retrievable | |  |
| Sepucha 2022 | | Did not evaluate any shared decision-making tool but ~~on~~ a SDM skills training intervention | | Wrong intervention | |  |
| Shokar 2010 | | No SDM tool was clearly defined in this study, and outcomes were not relevant to the SDM process or cognitive-affective outcome of an individual. | | Wrong context | |  |
| Sifri 2010 | | This study only explored factors associated with colorectal cancer screening decision stage and not on the effectiveness of an SDM tool. | | Wrong context | |  |
| Spring 2019 | | Involved hospitalized [symptomatic] participants in the study to test the effect of SDM intervention for lung cancer screening. | | Wrong population | |  |
| Starosta 2015 | | This study did not focus on evaluating the effectiveness of a decision aid but rather on the impact of attitudes about prostate cancer screening on the use of decision aid and screening. | | Wrong context | |  |
| Studts 2020 | | This study was conducted using an online survey and no description about the eligibility of participants for inclusion in the study was presented. | | Wrong study design | |  |
| Tabriz 2021 | | No vulnerable populations were included in this qualitative study. | | Wrong population | |  |
| Talcott 2016 | | This qualitative study did not include outcomes relevant to SDM tool preferences in terms of content, format, or delivery strategies. | | Wrong outcome | |  |
| Tan 2022 | | This is a cross-sectional study and does not involve a SDM tool intervention. | | Wrong context | |  |
| Tiedje 2021 | | Unable to determine the baseline characteristics of the study participants. | | Different population | |  |
| Tisnado 2015 | | This is a pilot testing and development study of a decision aids for mammography screening and not an effectiveness study. | | Wrong study design | |  |
| Toft 2019 | | No SDM tool presented in this study and outcome measures are not relevant to the SDM process nor to the cognitive-affective outcomes of participants. | | Wrong context | |  |
| Tomko 2015 | | This study did not include any comparators in the analyses and only measured the extent to which an interactive, web-based decision aid was associated with decisional and screening outcomes. | | Wrong study design | |  |
| Tomko 2015 | | This study did not include any relevant outcome on the SDM process and on the cognitive-affective outcome of an individual. | | Wrong outcome | |  |
| Tonge 2019 | | This study did not include any relevant SDM tool intervention as well as outcome measures on the preferred SDM tool’s content, format and/or delivery strategies for the qualitative review. | | Wrong outcome and intervention | |  |
| Yang 2020 | | This study did not involve any SDM tool but rather on physicians' approach in framing messages for mammography screening. | | Wrong intervention | |  |
| Yuen 2021 | | This qualitative study included less than 50% vulnerable populations. | | Different populations | |  |
| Valentine 2022 | | Focuses generally on the shared decision making (SDM) intervention instead of the effects of the SDM tool | | Wrong intervention | |  |
| Wang 2018 | | This study did not evaluate any SDM tool for cancer screening but rather on an intervention that trains clinicians to increase colorectal cancer screening. | | Wrong intervention | |  |
| Wangmar 2021 | | The study did not include and evaluated any SDM tool. | | Wrong intervention | |  |
| Watson 2006 | | This study was conducted and published before 2010. | | Year published before 2010 | |  |
| Weiner 2018 | | The study design is a randomized survey study, which is not a sufficient study design to measure effects of decision aids on SDM process and on the cognitive-affective outcomes of participants facing screening decisions. | | Wrong study design | |  |
| Weinstein 2019 | | This is a feasibility pilot study and not an effectiveness study on a SDM tool. | | Wrong study design | |  |
| Wood 2019 | | This is a pilot testing and development study of a tool to elicit women’s preferences for cervical cancer screening and not an effectiveness study. | | Wrong study design | |  |
| Woolf 2018 | | The study did not involve vulnerable populations for the qualitative review. | | Wrong population | |  |
| Wong 2015 | | This is a pilot-testing and development study and did not intend to investigate the effects on a shared decision-making tool for cancer screening. | | Wrong study design | |  |
| Wunderlich 2010 | | The study only evaluated the concordance of patient preference for SDM and its actual use in practice but did not specifically involve SDM tools. | | Wrong context | |  |
| Vaughan 2019 | | This is still a pilot and development study and not an effectiveness study of any SDM tool. | | Wrong study design | |  |
| Verma 2022 | | This study is more focused on the SDM intervention as a whole and not on the effects on the use of a SDM tool. | | Wrong intervention | |  |
| Volk 2008 | | This study was published before the year 2010. | | Published before 2010 | |  |
| Volk 2014 | | This is a pilot testing and development study design of an online case-based approach to SDM skills training for clinicians. | | Wrong study design | |  |
| Volk 2018 | | This study is an implementation report of a randomized controlled trial for patient-centered decision aid. | | Wrong context | |  |
| Zikmund-Fisher 2010 | | There is no well-defined SDM tool for cancer screening presented in this study. | | Wrong intervention | |  |
| Zhu 2022a | | There is no sufficient information on the involvement of an SDM tool and were more focused on associated factors of an informed decision. | | Wrong context | |  |
| Zhu 2022b | | This study did not involve and evaluated any SDM tool. | | Wrong intervention | |  |
|  |  | |  | |  | |
|  |  |  |  |  |  |  |
