# Supplementary material for: Mixed-Method Systematic Review and Meta-Analysis of Shared Decision-Making Tools for Cancer Screening
Source: Cancers (Basel). 2023 Jul 29;15(15):3867. doi: 10.3390/cancers15153867 (PMC10417450; doi:10.3390/cancers15153867)
Supplement: Supplementary file 1 [file cancers-15-03867-s001.zip › cancers-2455744-supplementary/Supplementary file_5_search strategy copy.docx]

**File S5. Search Strategies**

**Cochrane Library and EBM databases (via OVID)**

| # | **Searches** | **Results** |
| --- | --- | --- |
| **1** | ((exp decision-making/ or decision.ab,kf,ti. or decid*. ab,kf,ti. or decision-making.ab,kf,ti.) ADJ3 (shared.ab,kf,ti or collaborative.ab,kf,ti or patient*.ab,kf,ti. or parent*.ab,kf,ti. or carer*.ab,kf,ti. or caregiver*.ab,kf,ti. or care-giver*.ab,kf,ti. or guardian*.ab,kf,ti. or family support.ab,kf,ti.)) OR SDM.ab,ti,kw | 5816 |
| **2** | ((decision* or decid*) ADJ5 (discuss* or board or guide* or guidance or counseling or interact*)).ab,kf,ti. | 2559 |
| **3** | 3 (exp Risk Assessment/ or (risk-communication or risk-assessment or risk-information or individual risk or personalised risk or personalized risk or communicating risk or assessing risk or risk-presentation or risk-benefit information or Informed consent*).ab,kf,ti.) AND (patient or parent* or carer* or caregiver* or care-giver* or guardian* or family support).ti,ab,kf. | 32655 |
| **4** | (support*.ab,kf,ti. or aid.ab,kf,ti. Or aids.ab,kf,ti. or tool.ab,kf,ti. or tools.ab,kf,ti. or instrument*.ab,kf,ti. or technolog*.ab,kf,ti. or technique.ab,kf,ti. or techniques.ab,kf,ti. or system*.ab,kf,ti. or program*.ab,kf,ti. or algorithm*.ab,kf,ti. or process*.ab,kf,ti. or method*.ab,kf,ti. or intervention*.ab,kf,ti. or material*.ab,kf,ti. or computer*.ab,ti,kf) | 1299781 |
| **5** | 1 or 2 or 3 | 39999 |
| **6** | 5 AND 4 | 37937 |
| **7** | Early Detection of Cancer/ | 1645 |
| **8** | exp Mass screening/ or screen*.ti,ab,kf. | 87331 |
| **9** | exp Neoplasms/ or neoplasm*. ab,kf,ti. or cancer*.ab,kf,ti. or tumor*. ab,kf,ti. or carcinoma*.ab,kf,ti. Or Neoplasia*.ab,kf,ti.  or Malignanc*.ab,kf,ti. | 241303 |
| **10** | 8 AND 9 | 17402 |
| **11** | 7 or 10 | 17611 |
| **12** | **6 AND 11 (filtered by year: between 2010 to 2022)** | **1365** |

**EMBASE, and PUBMED (not MEDLINE) Database**

| # | **Searches** | **Results** |
| --- | --- | --- |
| **1** | 'decision'/exp OR decision OR decid*:ab,ti OR 'decision making'/exp OR 'decision making' | 898755 |
| **2** | Shared:ab,ti OR collaborative:ab,ti OR patient*:ab,ti OR parent*:ab,ti OR carer*:ab,ti OR caregiver*:ab,ti OR 'care giver*':ab,ti OR guardian*:ab,ti OR ‘family support’:ab,ti | 12.1M |
| **3** | #1 AND #2 | 442860 |
| **4** | 'risk assessment'/exp OR 'risk communication':ab,ti OR 'risk assessment':ab,ti OR 'risk information':ab,ti OR 'individual risk':ab,ti OR 'personalized risk':ab,ti OR 'communicating risk':ab,ti OR 'assessing risk':ab,ti OR 'risk presentation':ab,ti OR 'risk-benefit information':ab,ti OR 'informed consent':ab,ti | 826065 |
| **5** | Patient:ab,ti OR parent*:ab,ti OR carer*:ab,ti OR caregiver*:ab,ti OR 'care giver*':ab,ti OR guardian*:ab,ti OR 'family support':ab,ti | 4.8M |
| **6** | #4 AND #5 | 178369 |
| **7** | ((decision* or decid*) NEAR/5 (discuss* or board or guide* or guidance or counseling or interact*)):ab,ti | 46968 |
| **8** | support*:ab,ti OR aid:ab,ti OR aids.:ab,ti OR tool:ab,ti OR tools:ab,ti OR instrument*:ab,ti OR technolog*:ab,ti OR technique:ab,ti OR techniques:ab,ti OR system*:ab,ti OR program*:ab,ti OR algorithm*:ab,ti OR process*:ab,ti OR method*:ab,ti OR intervention*:ab,ti OR material*:ab,ti OR computer*:ab,ti | 20.2M |
| **9** | #3 OR #6 OR #7 | 624111 |
| **10** | #7 AND #8 | 516199 |
| **11** | 'early detection of cancer'/exp | 10775 |
| **12** | ('mass screening'/exp OR screen*:ab,ti) NOT (molecular:ab,ti OR genetic*:ab,ti OR snp:ab,ti) | 1.1M |
| **13** | 'neoplasms'/exp OR neoplasm*:ab,ti OR cancer*:ab,ti OR tumor*:ab,ti OR carcinoma*:ab,ti OR neoplasia*:ab,ti OR NOT (molecular:ab,ti OR genetic*:ab,ti OR snp:ab,ti) | 5.9M |
| **14** | #12 AND #13 | 298737 |
| **15** | #11 OR #14 | 303196 |
| **16** | **#10 AND #15** | 12123 |
| **17** | #10 AND #15 AND ([article]/lim OR [article in press]/lim OR [conference paper]/lim OR [preprint]/lim) AND [humans]/lim AND [english]/lim AND [abstracts]/lim AND ([embase]/lim OR [preprint]/lim OR [pubmed-not-medline]/lim) AND [2010-2022]/py | **3339** |

**Web of Science Advanced Search**

| # | **Searches** | **Results** |
| --- | --- | --- |
| **1** | **(((ALL=(decision)) OR TS=(decision)) OR TS=(decid*)) OR ALL=(decision making)** | 1946763 |
| **2** | ((((((((TS=(Shared)) OR TS=(collaborative)) OR TS=(patient*)) OR TS=(parent*)) OR TS=(carer*)) OR TS=(caregiver*)) OR TS=(care giver)) OR TS=(guardian*)) OR TS=(family support) | 9.1M |
| **3** | #1 AND #2 | 468627 |
| **4** | ((((((((((ALL=(risk assessment)) OR AB=(risk communication)) OR AB=(risk assessment)) OR AB=(risk information)) OR AB=(individual risk)) OR AB=(personalized risk communication)) OR AB=(communicating risk)) OR TS=(risk presentation)) OR TS=(risk-benefit information)) OR AB=(informed consent)) NOT AB=(prediction model OR risk prediction modeling OR risk prediction) | 1093830 |
| **5** | #2 AND #4 | 457145 |
| **6** | ((((ALL=(decision)) OR TS=(decid*)) OR TS=(discuss*)) AND TS=(disc)) AND TS=(board OR guide* OR guidance OR counseling OR interact*) | 1828 |
| **7** | TS=(support* OR aid OR aids OR tool OR tools OR instrument* OR technology* OR technique OR techniques OR system* OR program* OR algorithm* OR process* OR method* OR intervention* OR material* OR computer*) | 33.3M |
| **8** | #3 OR #5 OR #6 | 877314 |
| **9** | #7 AND #8 | 717098 |
| **10** | ALL=(early detection of cancer) | 61096 |
| **11** | ((ALL=(mass screening)) OR AB=(screen)) NOT TS=(molecular OR genetic* OR snps OR cell OR cells OR cellular OR biomarker* OR in vitro) | 598304 |
| **12** | ((((((ALL=(neoplasms)) OR TS=(neoplasm*)) OR TS=(cancer*)) OR TS=(tumor*)) OR TS=(carcinoma*)) OR TS=(neoplasia*)) NOT TS=(molecular OR genetic* OR snps OR cell OR cells OR cellular OR biomarker* OR in vitro) | 2085177 |
| **13** | #11 AND #12 | 5.9M |
| **14** | #10 OR #13 | 134128 |
| **15** | #9 AND #14 | 14028 |
| **16** | **Filters:**  **Review Article** (Exclude – Document Types) and **Early Access** or **Proceeding Paper** or **Article** (Document Types) and **1.111 Liver & Colon Cancer** or **1.119 Breast Cancer Scanning** or **1.199 Lung Cancer** or **1.147 Prostate Cancer** or **1.179 Oncology** or **1.273 Health Literacy & Telemedicine** or **1.155 Medical Ethics** or **4.61 Artificial Intelligence & Machine Learning** or **4.17 Computer Vision & Graphics** | **4011** |
