# Supplementary material for: Mixed-Method Systematic Review and Meta-Analysis of Shared Decision-Making Tools for Cancer Screening
Source: Cancers (Basel). 2023 Jul 29;15(15):3867. doi: 10.3390/cancers15153867 (PMC10417450; doi:10.3390/cancers15153867)
Supplement: Supplementary file 1 [file cancers-15-03867-s001.zip › cancers-2455744-supplementary/Supplementary file_3_Forest_Funnel plots_and_RoBs copy.docx]

**Supplementary file 3**

**A. Subgroup analysis**

**Figure S1.** Subgroup analysis on knowledge outcomes: (**A**) intervention duration of 6 months and below, and (**B**) above 6 months, (**C**) assessed using self-administered questionnaire, and (**D**) assessed using interview approach.

**
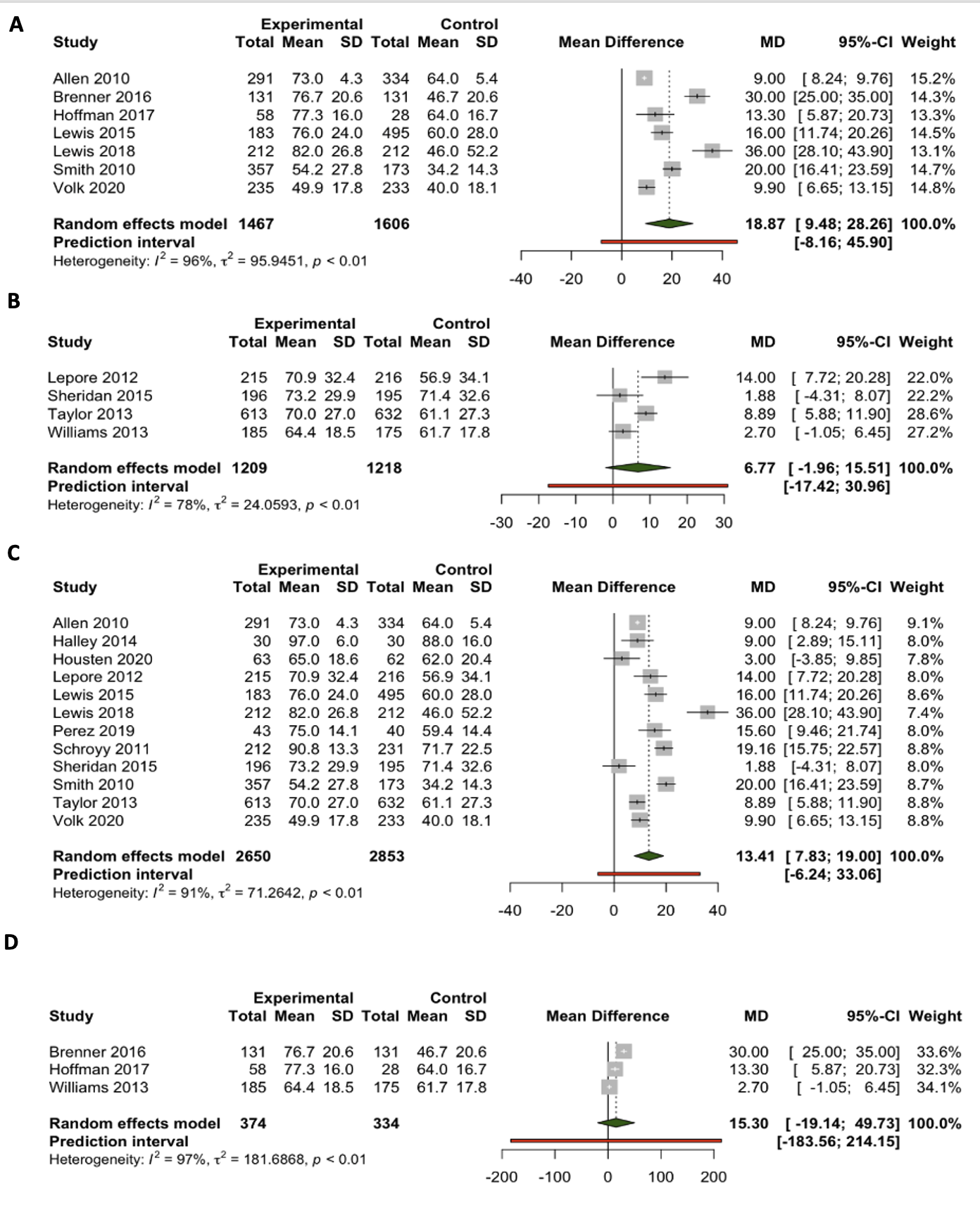
**

**Figure S2.** Subgroup analysis on knowledge outcomes based on targeted cancer screening: (**A**) colorectal cancer screening, (**B**) prostate cancer screening

**
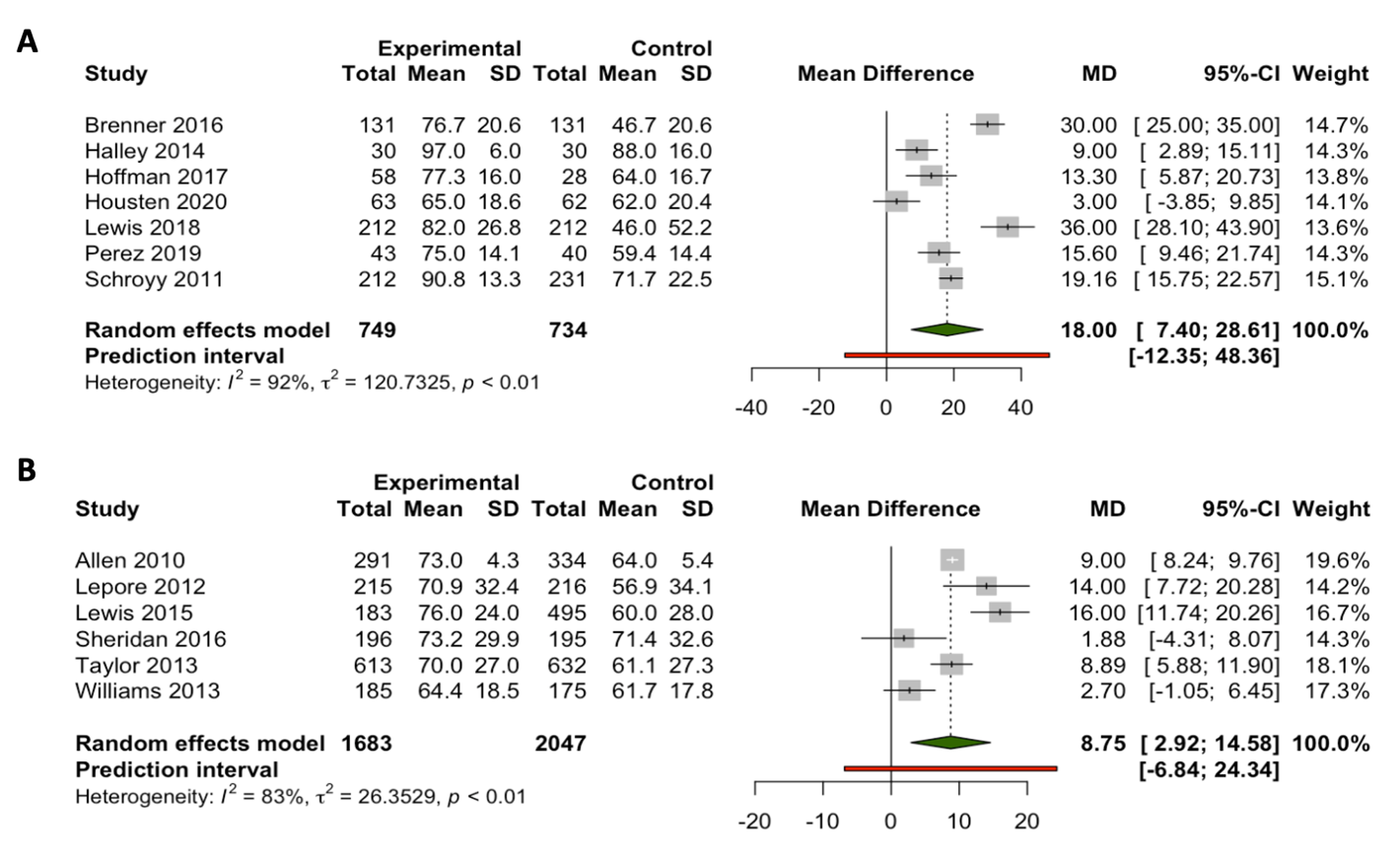
**

**Note:** All included in the meta-analysis for the knowledge outcomes have a randomized controlled trial study design

**Figure S3.** Subgroup analysis on decisional conflict outcomes: (**A**) RCT study designs, (**B**) before-after study designs, (**C**) SDM tools compared with usual care, and (**D**) SDM tools compared with attention controls or standard materials.

**
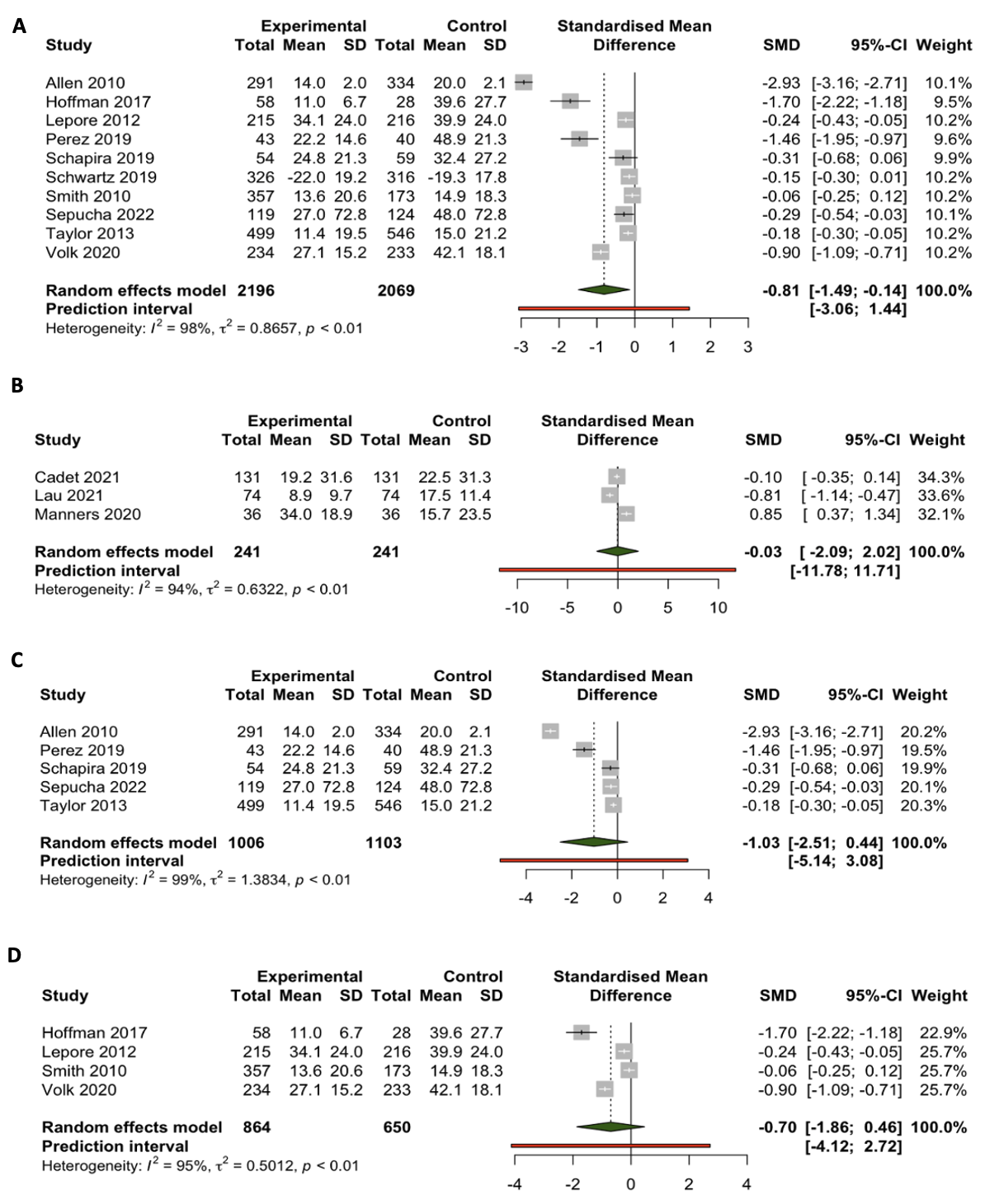
**

**Figure S4.** Subgroup analysis on decisional conflict outcomes: (**A**) for prostate cancer screening, (**B**) breast cancer screening, (**C**) colorectal cancer screening, and (**D**) lung cancer screening.

**
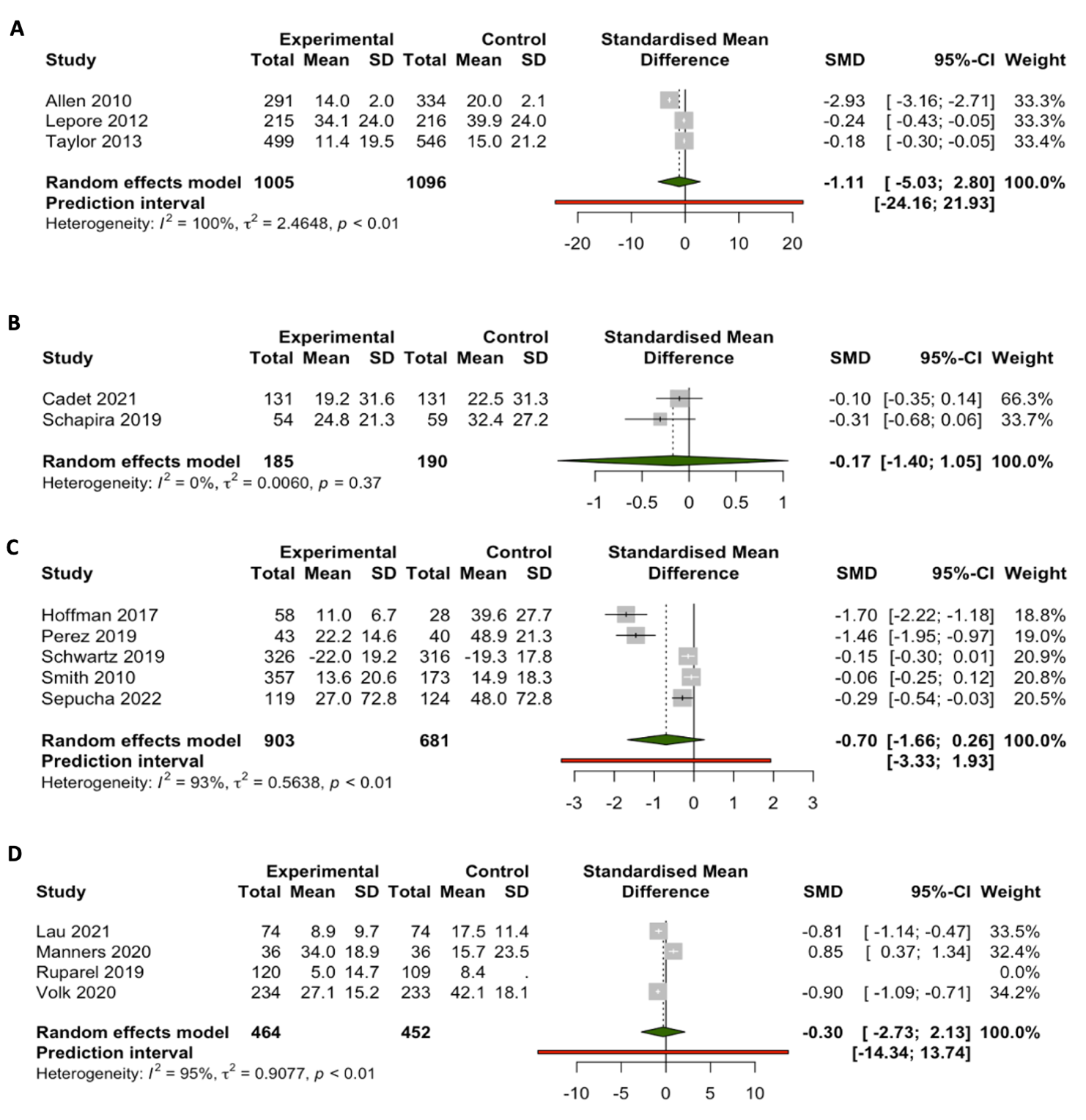
**

**Figure S5.** Subgroup analysis on intention to screen outcomes: (**A**) Randomized controlled trials and (**B**) before-after studies

**
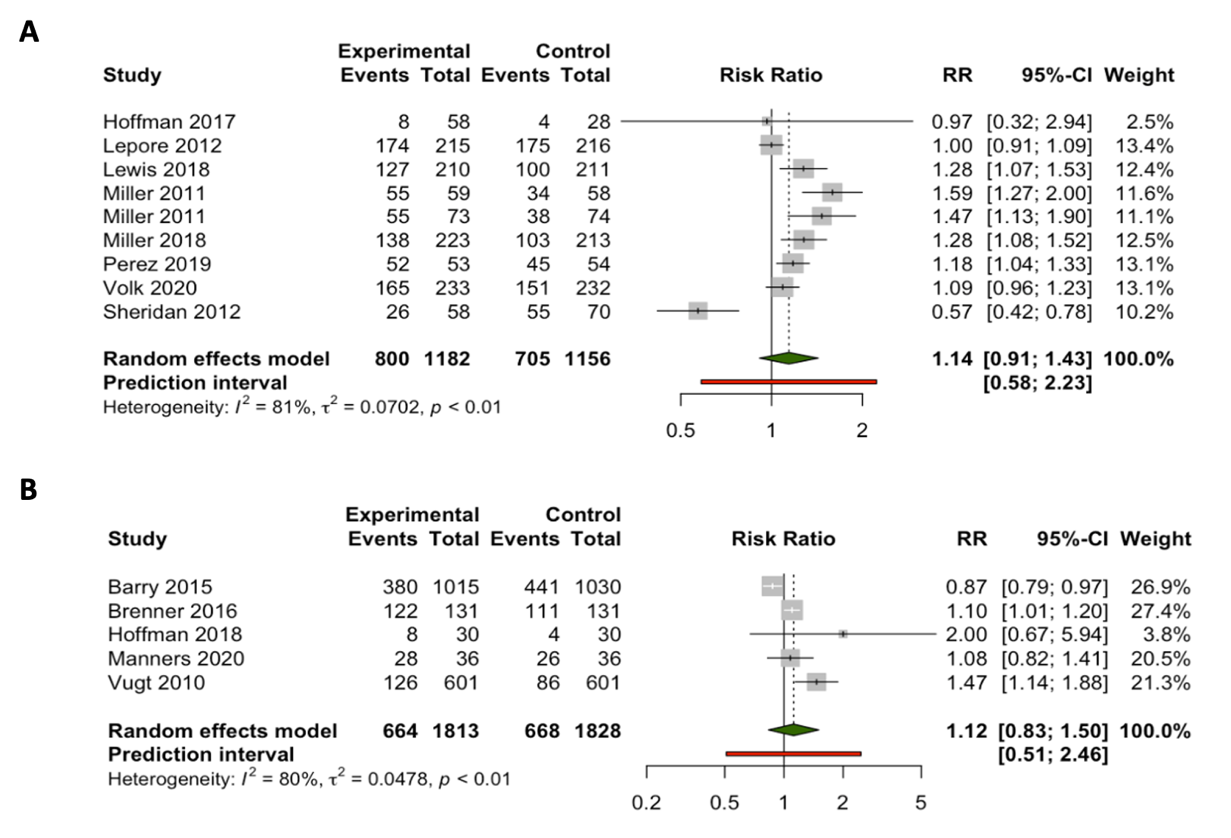
**

**Figure S6.** Subgroup analysis on intention to screen outcomes: (**A**) for prostate cancer screening, (**B**)colorectal cancer screening, and (**C**) lung cancer screening.

**
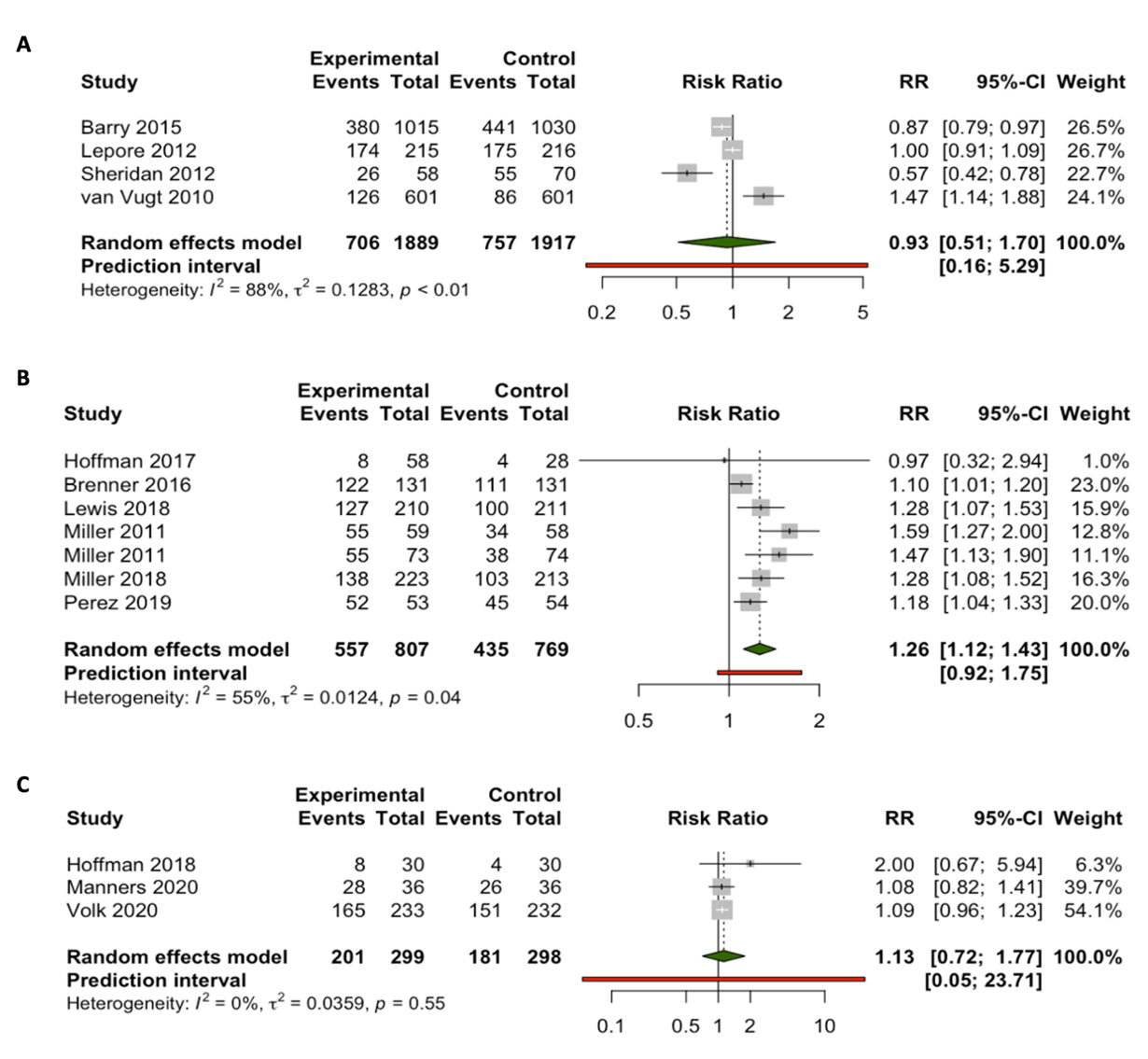
**

**B. PUBLICATION BIAS FOR KNOWLEDGE OUTCOME**

**Figure S7.** Forest plot for knowledge outcome (Standardized Mean Difference)

**
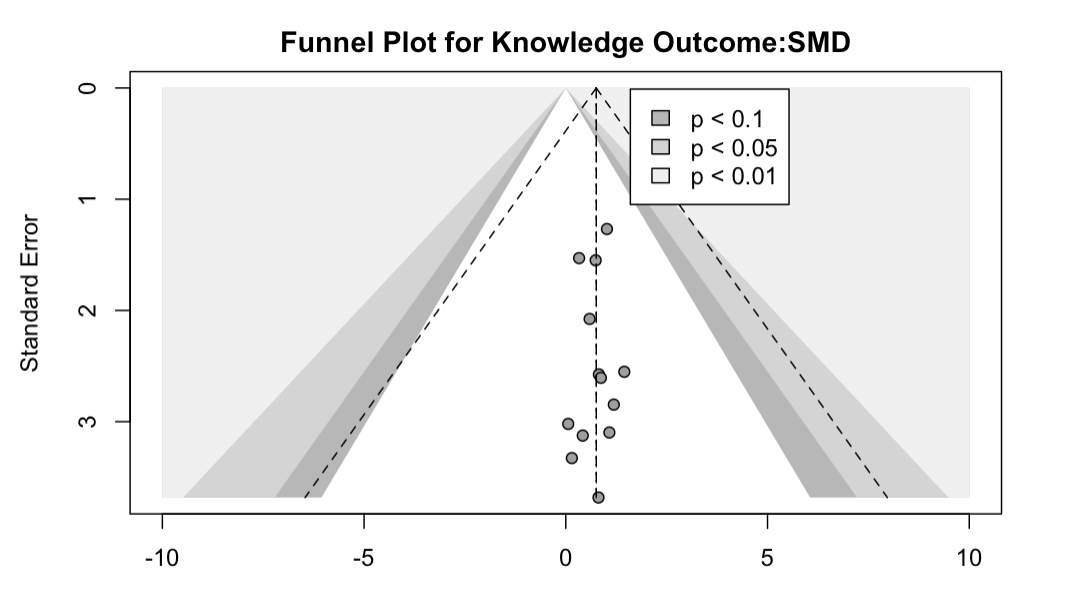
**

**Figure S8.** Forest plot for intention to screen (Relative Risk)

**
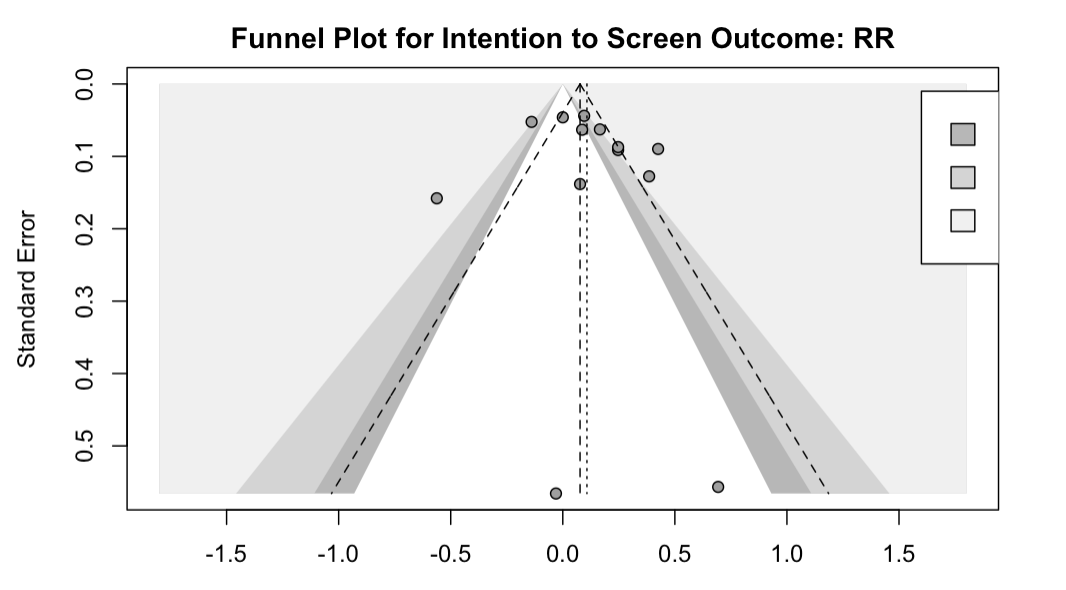
**

**C. CHARACTERISTICS OF INCLUDED STUDIES AND RISK OF BIAS ASSESSMENT** [ordered by study ID]

**Figure S9.** Risk of Bias Summary for Included Randomized Controlled Trial Studies


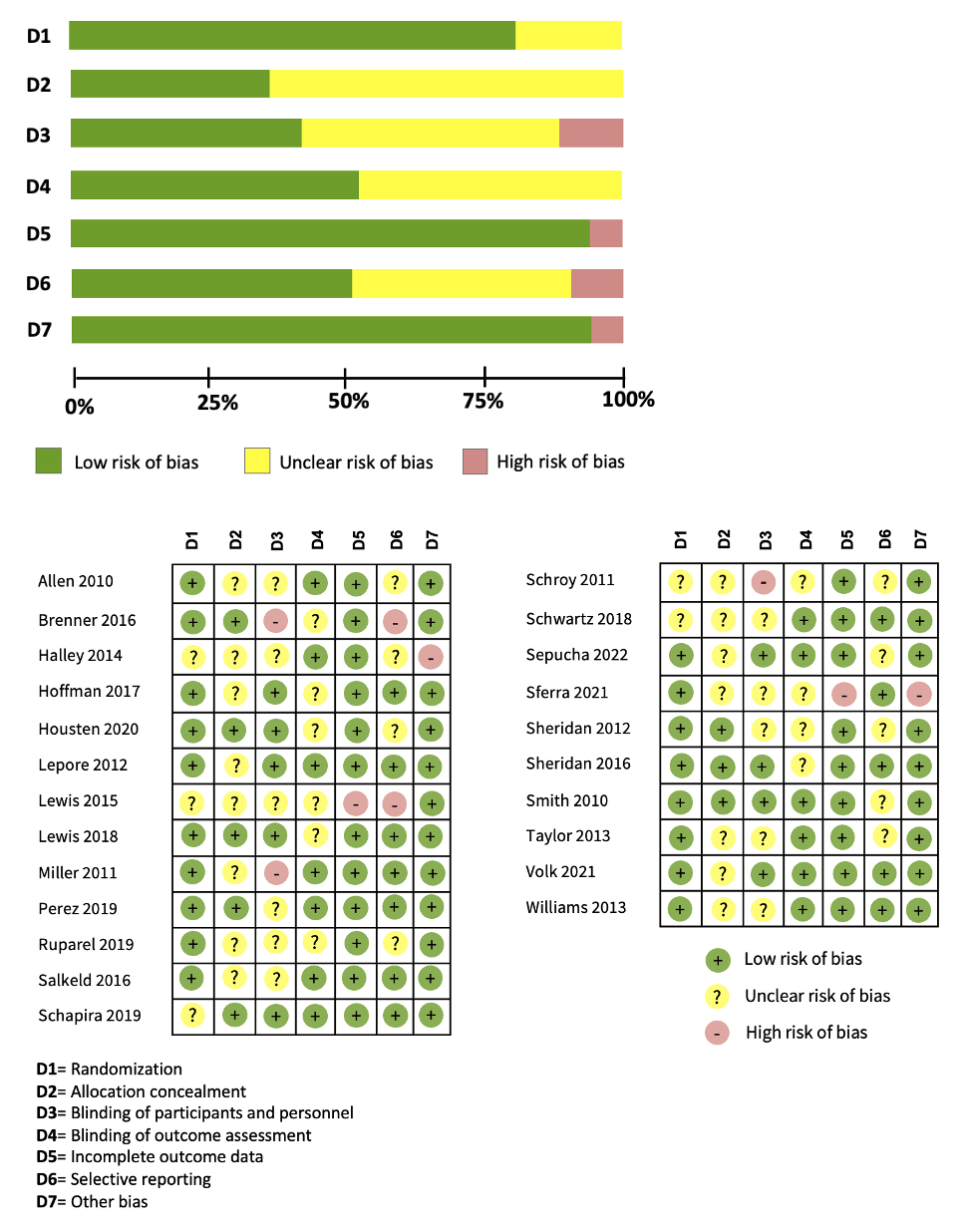


| **Allen 2010** | | |
| --- | --- | --- |
| Methods | Randomized to receive decision aid or non-intervention comparison | |
| Participants | 398 + 414 participants facing preference-sensitive decision on prostate cancer screening | |
| Interventions | DA titled “The PSA Test: Is it Right for You?” computer-tailored DA  Comparator: no intervention | |
| Outcomes | Primary: intention to screen, readiness to decide, and preferred role in decision making  Secondary: Knowledge about PSA screening | |
| Notes | **-** | |
| **Risk of Bias** | **Author’s judgement** | **Support for judgement** |
| Random sequence generation  (selection bias) | Low risk | Mentioned the use of computer-generated random numbers to condition within blocks for the randomization (p 3, METHODS: Settings section) |
| Allocation concealment  (selection bias) | Unclear risk | No sufficient information to make a judgement |
| Blinding of participants and personnel (performance bias) All outcomes | Unclear risk | No sufficient information to make a judgement |
| Blinding of outcome assessment (detection bias) | Low risk | Outcomes were measured objectively using validated questionnaires and were self-administered. Thus, responses were not influenced by researchers (p 6, Measures: Primary Outcomes and Secondary Outcomes sections). |
| Incomplete outcome data  (attrition bias) | Low risk | No incomplete or missing data found and attrition was very low for both arms. |
| Selective reporting  (reporting bias) | Unclear risk | The study did not mention any specific details or indications of selective reporting. |
| Other bias | Low risk | No other potential bias found. |

| **Brenner 2016** | | |
| --- | --- | --- |
| Methods | Randomized to either the CRC screening decision aid or the control video | |
| Participants | 134+133 vulnerable patients considering colorectal cancer screening | |
| Interventions | CRC screening DA: OPCIONES and CHOICE DA  Comparator: Usual care | |
| Outcomes | CRC related knowledge, discussion, test preference, intent to be screened, and test ordering | |
| Notes | - | |
| **Risk of Bias** | **Author’s judgement** | **Support for judgement** |
| Random sequence generation  (selection bias) | Low risk | Use of permuted blocks in randomization (shown in another reports, citation presented in reference #41 of Brenner 2016 report). |
| Allocation concealment  (selection bias) | Unclear risk | Use of sequentially sealed, opaque envelops that contained randomly assigned intervention (reported in their protocol) |
| Blinding of participants and personnel (performance bias) All outcomes | High risk | Reported that no blinding of research staff to the participants’ assigned study arm and did not present approaches to reduce performance bias (p 8, Limitations) |
| Blinding of outcome assessment (detection bias) | Unclear risk | Information not provided |
| Incomplete outcome data  (attrition bias) | Low risk | Reported only 2% loss to follow-up (p 8, limitations) |
| Selective reporting  (reporting bias) | High risk | Reported screening communication and test ordering outcomes are patient-reported, which may result to inaccurate reporting (p 8, Limitations) |
| Other bias | Low risk | No other bias found |

| **Halley 2014** | | |
| --- | --- | --- |
| Methods | Randomized to receive web-first  or DVD-first decision aid and conducted one-on-one interview with the patients to explore patients preference in terms of DA’s format | |
| Participants | Included patients considering colorectal cancer screening | |
| Interventions | Intervention: Web-first DA  Comparator: DVD-first DA | |
| Outcomes | Knowledge, web utilization (quantitative), patient’s format preference | |
| Notes | This is mixed-method study. | |
| **Risk of Bias** | **Author’s judgement** | **Support for judgement** |
| Random sequence generation (selection bias) | Unclear risk | No sufficient information to make a  judgment |
| Allocation concealment  (selection bias) | Unclear risk | No information provided |
| Blinding of participants and personnel (performance bias)  All outcomes | Unclear risk | No information provided |
| Blinding of outcome assessment (detection bias) | Low risk | Did not mention about blinding of outcome but the outcome were obtained objectively for the knowledge outcome and via tracking software for the web utilization. |
| Incomplete outcome data  (attrition bias) | Low risk | No missing or incomplete data found. |
| Selective reporting (reporting bias) | Unclear risk | The study protocol mentioned in the report was not very clear. No sufficient information to make judgement. |
| Other bias | High risk | Mentioned to mostly include highly educated, high-income, well-insured individuals (p 2890, Discussion section) (potential selection bias). |

| **Hoffman 2017** | | |
| --- | --- | --- |
| Methods | Randomized to view either a patient decision aid video or an attention control video | |
| Participants | African American patients considering colorectal cancer screening | |
| Interventions | PtDA: video-based culturally-tailored PtDA using an entertainment-education formation  Comparator: Attention-control video about hypertension | |
| Outcomes | Patient’s knowledge, decisional conflict, self-advocacy, attitudes, perceived social norms, and intentions to screen and completion of screening. | |
| Notes | **-** | |
| **Risk of Bias** | **Author’s judgement** | **Support for judgement** |
| Random sequence generation (selection bias) | Low risk | Mentioned the use of computer-generated permuted blocks in a two-to-one ratio for randomization |
| Allocation concealment  (selection bias) | Unclear risk | No sufficient information to make a judgement |
| Blinding of participants and personnel (performance bias)  All outcomes | Low risk | Mentioned blinding of participants and interviewers until baseline questionnaires were completed. |
| Blinding of outcome assessment (detection bias) | Unclear risk | No information provided |
| Incomplete outcome data  (attrition bias) | Low risk | Attrition was very low |
| Selective reporting (reporting bias) | Low risk | Mentioned registration of protocol and conduct of trials in the report |
| Other bias | Low risk | No other bias found |

| **Housten 2020** | | |
| --- | --- | --- |
| Methods | Randomized to view either  the audio booklet, static video or the animated video | |
| Participants | Patients with low health literacy considering colorectal cancer screening | |
| Interventions | Intervention: animated video  Comparators: audio booklet and static video | |
| Outcomes | Total knowledge, verbatim knowledge and Gist knowledge | |
| Notes | **-** | |
| **Risk of Bias** | **Author’s judgement** | **Support for judgement** |
| Random sequence generation (selection bias) | Low risk | Reported to use a computer generated  permuted-block randomization (p 4, Randomization and Study Procedures section) |
| Allocation concealment  (selection bias) | Low risk | Reported to use a sealed randomization assignment envelope (p 4, Randomization and Study Procedures section) |
| Blinding of participants and personnel (performance bias)  All outcomes | Low risk | Mentioned the use of double-blind procedure to 1 of the 3 intervention arms (p 4, Randomization and Study Procedures section) |
| Blinding of outcome assessment (detection bias) | Unclear risk | No sufficient information to make a judgement |
| Incomplete outcome data  (attrition bias) | Low risk | No missing data were observed. |
| Selective reporting (reporting bias) | Unclear risk | A study protocol was mentioned but it was not clear whether the protocol is accessible (p 4, Methods: Study Participants section). No sufficient information to make judgement. |
| Other bias | Low risk | No other potential bias observed |

| **Lepore 2012** | | |
| --- | --- | --- |
| Methods | Randomized to receive either a decision support intervention or attention control material | |
| Participants | Men considering prostate cancer testing | |
| Interventions | Decision support intervention in Pamphlet format with tailored telephone education about prostate cancer testing  Comparators:  fruit and vegetable consumption attention control | |
| Outcomes | Knowledge about prostate cancer and testing, decisional conflict, testing intention, benefits-to-risk ration of testing, verified PSA testing, congruence between intention to get tested and actual testing, and anxiety | |
| Notes | **-** | |
| **Risk of Bias** | **Author’s judgement** | **Support for judgement** |
| Random sequence generation (selection bias) | Low risk | Reported the use of computer-generated randomization schedule (page 4, Enrollment and Sample size calculations section). |
| Allocation concealment  (selection bias) | Unclear risk | No sufficient information to make a judgement |
| Blinding of participants and  personnel (performance bias)  All outcomes | Low risk | Data collectors were blinded to condition but interventionist were not as it is not possible to blind them. However, interventionists were trained at conducting telephone calls page 4, Enrollment and Sample size calculations section). |
| Blinding of outcome assessment (detection bias) | Low risk | The report generator was blinded to participant’s study condition and used validated questionnaires to assess outcome measures ((page 6-7, Measures section). |
| Incomplete outcome data  (attrition bias) | Low risk | Reported to have no missing data at pretest and missing data at posttest were limited only to self-reports of men lost to follow-up (12%). Nonetheless, there were no significant difference between participants who were and were not lost to follow-up on outcome measures and conducted sensitivity analyses (Page 8, Analytic Strategy section). |
| Selective reporting (reporting bias) | Low risk | No evidence of selective reporting observed. |
| Other bias | Low risk | No other bias found |
| **Lewis 2015** | | |
| Methods | Randomized to receive either a decision support intervention or ~~vs.~~ usual care | |
| Participants | Men considering prostate cancer screening | |
| Interventions | Decision support intervention (DESI) in DVD format + invitation to participate in shared (group) medical appointment (SMA)  Comparators:  DESI only, SDA invitation only and no intervention material/usual care | |
| Outcomes | DESI viewing, Screening utilization at 12 months post-mailing, PSA decision-specific knowledge, and attitude towards PSA testing | |
| Notes | **-** | |
| **Risk of Bias** | **Author’s judgement** | **Support for judgement** |
| Random sequence generation (selection bias) | Unclear risk | No sufficient information to make a judgement |
| Allocation concealment  (selection bias) | Unclear risk | No sufficient information to make a judgement |
| Blinding of participants and  personnel (performance bias)  All outcomes | Unclear risk | No information provided |
| Blinding of outcome assessment (detection bias) | Unclear risk | No information provided |
| Incomplete outcome data  (attrition bias) | High risk | Reported to have 23% of the participants that opted-out at 4 months follow-up and the proportion of participants that viewed DESI were low and were self-selected. |
| Selective reporting (reporting bias) | High risk | No protocol presented and important findings for some outcomes were not clearly reported, particularly the number of participants who responded to survey on change of knowledge pre- and post-viewing of DESI+SMA (p 814, table 2). |
| Other bias | Low risk | No other bias found |

| **Lewis 2018** | | |
| --- | --- | --- |
| Methods | Randomized to receive either a decision aid or to attention control | |
| Participants | 212+ 212 older adults considering colon cancer screening | |
| Interventions | DA: paper-based patient decision aid  Control | |
| Outcomes | Appropriate screening behavior and intent  Knowledge, value clarity, preparedness for individualized decision-making, patient screening preference prior to provider visit, CRC screening discussion occurred (SDM), patient initiated screening discussion | |
| Notes | **-** | |
| **Risk of Bias** | **Author’s judgement** | **Support for judgement** |
| Random sequence generation (selection bias) | Low risk | Mentioned the use of a centralized computer process- permuted blocks stratified by healthcare, for intervention randomization (p 616, Randomization and Blinding section) |
| Allocation concealment (selection bias) | Low risk | Mentioned the use to opaque sealed envelopes to conceal allocation of interventions (p 616, Randomization and Blinding section) |
| Blinding of participants and personnel (performance bias)  All outcomes | Low risk | Mentioned blinding of participants to their assignment (p 616, Randomization and Blinding section) |
| Blinding of outcome assessment (detection bias) | Unclear risk | No sufficient information provided to make a judgement |
| Incomplete outcome data (attrition bias) | Low risk | No incomplete or missing outcome data |
| Selective reporting (reporting bias) | Low risk | A protocol was provided and no selective reporting is observed. |
| Other bias | Low risk | No potential bias observed. |

| **Miller 2011** | | |
| --- | --- | --- |
| Methods | Randomized to receive either a web-based decision aid or a control program | |
| Participants | Mixed-literacy patients overdue for colorectal cancer screening | |
| Interventions | DA: Web-based DA  Comparator: control program before a scheduled primary care appointment | |
| Outcomes | Ability to state a screening test preference, readiness to receive screening, test ordering, and test completion | |
| Notes | **-** | |
| **Risk of Bias** | **Author’s judgement** | **Support for judgement** |
| Random sequence generation (selection bias) | Low risk | Mentioned the use of blocked randomization stratified by literacy level. |
| Allocation concealment (selection bias) | Unclear risk | No sufficient information provided to make a judgement. |
| Blinding of participants and personnel (performance bias)  All outcomes | High risk | Mentioned in the limitation section. No blinding of research assistants who administered the questionnaires. |
| Blinding of outcome assessment (detection bias) | Low risk | Mentioned the blinding of outcome assessors (p 7, Discussion section) |
| Incomplete outcome data (attrition bias) | Low risk | No incomplete or missing outcome data. |
| Selective reporting (reporting bias) | Low risk | Mentioned the approved protocol (p 2, Methods section). |
| Other bias | Low risk | No other potential bias observed. |

| **Perez 2019** | | |
| --- | --- | --- |
| Methods | Randomized to receive either a DA or usual care | |
| Participants | Latinos with limited-proficiency considering colorectal cancer screening | |
| Interventions | DA: web format DA  Comparator: usual care | |
| Outcomes | Decisional conflict, Knowledge of colorectal cancer and screening options, intention to screen, and attributed importance for eight characteristics of screening | |
| Notes | **-** | |
| **Risk of Bias** | **Author’s judgement** | **Support for judgement** |
| Random sequence generation (selection bias) | Low risk | Reported to use computer-based simple randomization (p 2, Methods section). |
| Allocation concealment (selection bias) | Low risk | Mentioned the use of a centralized off-site computer allocation process (blinding of allocation) by the researcher who recruited participants via phone call (p 2, Methods section). |
| Blinding of participants and personnel (performance bias)  All outcomes | Unclear risk | No sufficient information provided to make a judgement. |
| Blinding of outcome assessment (detection bias) | Low risk | Mentioned that researchers did not mediate in the participant’s responses to the questionnaires (p 2, Methods section). |
| Incomplete outcome data (attrition bias) | Low risk | No missing data was observed in the report |
| Selective reporting (reporting bias) | Low risk | Mentioned study protocol approved by an Ethics committee. No selective reporting observed. |
| Other bias | Low risk | Appears to have no other potential bias |

| **Ruparel 2019** | | |
| --- | --- | --- |
| Methods | Randomized to receive either an information film with booklet or a booklet alone | |
| Participants | 120 + 109 participants considering lung cancer screening (LCS) | |
| Interventions | DA: information film + information booklet  Comparator: information booklet alone | |
| Outcomes | Total knowledge about the benefits and harms of LCS, decisional conflict, and acceptability of the film and information booklet | |
| Notes | **-** | |
| **Risk of Bias** | Author’s judgement | Support for judgement |
| Random sequence generation (selection bias) | Low risk | Mentioned the use of simple parallel randomization without restriction (p 745, Study design and intervention section) |
| Allocation concealment  (selection bias) | Unclear risk | The study did not mention any specific details or indications of allocation concealment. |
| Blinding of participants and personnel (performance bias)  All outcomes | Unclear risk | No information provided |
| Blinding of outcome assessment (detection bias) | Unclear risk | No information provided |
| Incomplete outcome data  (attrition bias) | Low risk | Missing outcome data is observed to be very low. |
| Selective reporting (reporting bias) | Unclear risk | Mentioned to be included in a wider randomized trial but no protocol was reported. |
| Other bias | Low risk | No other bias were observed. |

| **Salkeld 2016** | | |
| --- | --- | --- |
| Methods | Randomized to receive either a personalized decision aid or a standard decision aid | |
| Participants | 727 + 720 men considering PSA screening | |
| Interventions | Intervention: Personalised DA with choice over the inclusion of up to 10 attributes  Comparator: Standard DA with a fixed set of five attributes | |
| Outcomes | Emergent opinion to have a PSA test or not, self-rated decision quality after completion of online DA, intention to screen in the next 12 months, and use of extra decision attributes | |
| Notes | **-** | |
| **Risk of Bias** | **Author’s judgement** | **Support for judgement** |
| Random sequence generation (selection bias) | Low risk | Mentioned the use of computerized randomization to the active comparator or active intervention group (p 5, Method- Phase 1: Developing a personalized decision aid for PSA screening section) |
| Allocation concealment  (selection bias) | Unclear risk | No sufficient information provided to make a judgement |
| Blinding of participants and  personnel (performance bias)  All outcomes | Unclear risk | No sufficient information provided to make a judgement |
| Blinding of outcome assessment (detection bias) | Low risk | No mention of blinding of outcome assessment, however, participants where the ones responsible for weighting the criteria of decision quality and all outcomes were measured objectively. |
| Incomplete outcome data  (attrition bias) | Low risk | No incomplete outcome data as well as attrition |
| Selective reporting (reporting bias) | Low risk | Study protocol was clearly reported. No selective reporting observed. |
| Other bias | Low risk | No other potential bias observed |

| **Schapira 2018** | | |
| --- | --- | --- |
| Methods | Randomized to receive either a patient decision aid (PtDA) or usual care | |
| Participants | 104+ 103 women considering breast cancer screening (BCS) | |
| Interventions | DA: PtDA with risk estimator  Comparator: Usual Care | |
| Outcomes | Intention to screen, Knowledge on BCS, and Decisional conflict | |
| Notes | - | |
| **Risk of Bias** | **Author’s judgement** | **Support for judgement** |
| Random sequence generation (selection bias) | Unclear | No sufficient information provided to make a judgement |
| Allocation concealment  (selection bias) | Low risk | Mentioned the concealment of assignment (p 3, Study protocol section) |
| Blinding of participants and  personnel (performance bias)  All outcomes | Low risk | Mentioned the blinding of research assistants to the outcomes that were conducting the review chart (p 3, Study protocol section) |
| Blinding of outcome assessment (detection bias) | Low risk | Mentioned the blinding of research assistants to the outcomes that were conducting the review chart (p 3, Study protocol section) |
| Incomplete outcome data  (attrition bias) | Low risk | No missing outcome data and attrition was very low. |
| Selective reporting (reporting bias) | Low risk | Clear description of the study protocol was reported. |
| Other bias | Low risk | No other potential bias observed. |

| **Schroy 2011** | | |
| --- | --- | --- |
| Methods | Comparison between either a DA+ risk assessment tool, DA alone or usual care | |
| Participants | Average-risk patients belonging to vulnerable populations that were considering colorectal cancer screening | |
| Interventions | Two Intervention-arm: DA+ “Your Disease Risk (YDR)” risk assessment tool OR DA alone  Comparator: Generic information entitled “9 Ways to Stay Healthy and Prevent Disease” | |
| Outcomes | Patient preference, knowledge, satisfaction with decision making process(SDMP), screening intentions, and test concordance | |
| Notes | Meta-analyses:  - Three-arm intervention, we only compared DA+ risk assessment tool vs. control for the meta-analyses.  - Scores were converted to percentage scores, dividing the actual score from exposure to each intervention by the total score (12) and multiplying it to 100. | |
| **Risk of Bias** | **Author’s judgement** | **Support for judgement** |
| Random sequence generation (selection bias) | Unclear risk | Insufficient information provided to make a judgement |
| Allocation concealment  (selection bias) | Unclear risk | No information provided |
| Blinding of participants and  personnel (performance bias)  All outcomes | High risk | Reported that the lack of blinding of providers may present bias on the intervention’s effect on the outcomes measured, particularly the SDMP outcome (p 13, Discussion section, 3^rd^ paragraph). |
| Blinding of outcome assessment (detection bias) | Unclear risk | No information provided |
| Incomplete outcome data  (attrition bias) | Low risk | All outcomes measured included have complete data/follow-up (p 18, Figure 2). |
| Selective reporting (reporting bias) | Unclear risk | Only reported to exclude the data on patient adherence in the interim analysis due to incomplete follow-up but this information is not sufficient enough to make judgement. |
| Other bias | Low risk | No other bias that can be identified by the review authors. |

| **Schwartz 2018** | | |
| --- | --- | --- |
| Methods | Randomized to receive either a verbal DA or a quantitative DA | |
| Participants | 364 + 364 primary care patients due for CRC screening | |
| Interventions | Intervention: quantitative decision aid  Comparator: verbal decision aid | |
| Outcomes | Perceived CRC risk, screening intent, test preference, and screening up- take at 6 months | |
| Notes | - | |
| **Risk of Bias** | **Author’s judgement** | **Support for judgement** |
| Random sequence generation (selection bias) | Unclear risk | Only mentioned the use of REDCap database to randomize participants only (p 727, 2.4. Study Procedure). |
| Allocation concealment  (selection bias) | Unclear risk | No sufficient information provided to make a judgement. |
| Blinding of participants and  personnel (performance bias)  All outcomes | Unclear risk | No sufficient information provided to make a  judgement. |
| Blinding of outcome assessment (detection bias) | Low risk | No information on blinding of outcome but outcomes were assessed objectively without the influence of research assistants. |
| Incomplete outcome data (attrition bias) | Low risk | Very low missing (< 5%) outcome data and attrition |
| Selective reporting (reporting bias) | Low risk | Protocol was reported (p 727, 2.1 Study setting section. |
| Other bias | Low risk | No further bias observed. |

| **Sepucha 2022** | | |
| --- | --- | --- |
| Methods | Randomized to receive either a decision worksheet or usual care | |
| Participants | 400 + 400 participants considering CRC screening using colonoscopy | |
| Interventions | Intervention: 3-pafe decision worksheet plus telephone session with a trained decision coach  Comparator: Usual care | |
| Outcomes | Screening uptake, SDM process, decisional conflict, screening preference, patient-healthcare provider communication in the past 2 months | |
| Notes | - | |
| **Risk of Bias** | **Author’s judgement** | **Support for judgement** |
| Random sequence generation (selection bias) | Low risk | Mentioned the use of a computer random number generator to assign in the intervention or control group (p 3, Randomization and blinding section) |
| Allocation concealment  (selection bias) | Unclear risk | No sufficient information provided to make a judgement |
| Blinding of participants and  personnel (performance bias)  All outcomes | Low risk | Blinding of staff who entered the data from the paper surveys and those who conducted chart review to collect screening (p 3, Randomization and blinding section). |
| Blinding of outcome assessment (detection bias) | Low risk | Reported no blinding of statisticians who were analyzing the results. Yet outcomes were assessed objectively. |
| Incomplete outcome data  (attrition bias) | Low risk | No incomplete outcome data and very low attrition rate (< 1%) |
| Selective reporting (reporting bias) | Unclear risk | The study did not mention any specific details or indications of selective reporting. |
| Other bias | Low risk | No other potential bias observed |

| **Sferra 2021** | | |
| --- | --- | --- |
| Methods | Randomized to receive either an Option Grid or a shouldiscreen.com DA | |
| Participants | 128 + 109 patients considering lung cancer screening | |
| Interventions | Interventions: SDM with Option Grid vs. SDM with Shouldiscreen.com DAs | |
| Outcomes | Decision regret, SDM process and knowledge of lung cancer screening | |
| Notes | - | |
| **Risk of Bias** | **Author’s judgement** | **Support for judgement** |
| Random sequence generation (selection bias) | Low risk | Mentioned the use of a web-based randomization tool to receive either of the interventions. |
| Allocation concealment  (selection bias) | Unclear risk | No sufficient information provided to make a judgement. |
| Blinding of participants and  personnel (performance bias)  All outcomes | Unclear risk | No sufficient information provided to make a judgement. |
| Blinding of outcome assessment (detection bias) | Unclear risk | No sufficient information provided to make a judgement. |
| Incomplete outcome data  (attrition bias) | High risk | Mentioned high non-response or incomplete response to the questionnaires. |
| Selective reporting (reporting bias) | Low risk | Presented the study protocol in the report. |
| Other bias | High risk | Did not measure the outcome measures at baseline, thus, mean difference between the interventions are hard to compare and evaluate. |

| **Sheridan 2012** | | |
| --- | --- | --- |
| Methods | Randomized to receive either the video-based DA, a researcher-led coaching session or a highway safety video | |
| Participants | 70 + 58 men considering prostate cancer screening and 28 physicians under one of the 4 practices | |
| Interventions | Intervention: video-based DA  Comparator: researcher-led coaching session or highway safety video | |
| Outcomes | Men’s perception that screening is a decision, knowledge on prostate cancer screening, self-reported participation in SDM, participation preference, screening intent and actual screening rates | |
| Notes | - | |
| **Risk of Bias** | **Author’s judgement** | **Support for judgement** |
| Random sequence generation (selection bias) | Low risk | Mentioned the use of a computer-generated random numbers for randomization (p 5, Intervention and survey delivery and the procedure of randomization section). |
| Allocation concealment  (selection bias) | Low risk | Reported to use sealed opaque envelopes to conceal the assigned random intervention (p 5, Intervention and survey delivery and the procedure of randomization section). |
| Blinding of participants and  personnel (performance bias)  All outcomes | Unclear risk | No sufficient information to make a judgement. |
| Blinding of outcome assessment (detection bias) | Unclear risk | No sufficient information to make a judgement. |
| Incomplete outcome data  (attrition bias) | Low risk | No incomplete outcome data/ attrition observed. |
| Selective reporting (reporting bias) | Unclear risk | No study protocol reported. |
| Other bias | Low risk | No other potential bias observed. |

| **Sheridan 2015** | | |
| --- | --- | --- |
| Methods | Randomized to receive either of the different formats of DA | |
| Participants | 195 + 192 + 196 + 192 patients considering low-value screening services, including prostate and colorectal cancer screening | |
| Interventions | Interventions: Narrative and Framed format DA  Comparators: Words and Numbers format DA | |
| Outcomes | Intention to screen, knowledge on prostate or colorectal cancer screening, and value clarity | |
| Notes | - | |
| **Risk of Bias** | **Author’s judgement** | **Support for judgement** |
| Random sequence generation (selection bias) | Low risk | Mentioned the use of a central computerized randomization to one of the 4 formats (p 32, Procedures section). |
| Allocation concealment  (selection bias) | Low risk | Mentioned allocation concealment conducted from staff in a computerized database until after completion of the baseline survey (p 32, Procedures section). |
| Blinding of participants and  personnel (performance bias)  All outcomes | Low risk | Mentioned blinding of participants by only informing them about “participating in a study on how to best communicate with patients about screening” (p 32, Procedures section). |
| Blinding of outcome assessment (detection bias) | Unclear risk | No sufficient information provided to make a judgement. |
| Incomplete outcome data  (attrition bias) | Low risk | All outcome data are complete. |
| Selective reporting (reporting bias) | Low risk | Presented the study protocol in the report. |
| Other bias | Low risk | No other potential bias observed. |

| **Smith 2010** | | |
| --- | --- | --- |
| Methods | Randomized to receive either a DA with question prompts, a DA with DVD, or standard information | |
| Participants | 196 + 188 + 188 adults with low educational attainment and literacy considering bowel cancer screening | |
| Interventions | Intervention: DA with question prompts or with DVD  Comparator: standard information booklet | |
| Outcomes | Informed choice (Adequate knowledge and consistency between attitudes and screening behavior), and preference for involvement in screening decisions | |
| Notes | - | |
| **Risk of Bias** | **Author’s judgement** | **Support for judgement** |
| Random sequence generation (selection bias) | Low risk | Mentioned the use of random drawing from the electoral register using the Australian Bureau of Statistics SEIFA codes as well as via random permuted blocks of size 6 and 9 stratified by sex (p 2-3, Participants and Recruitment section). |
| Allocation concealment (selection bias) | Low risk | Reported the use of a computer-assisted telephone interviewing system (p 2, Participants and Recruitment section). |
| Blinding of participants and personnel (performance bias)  All outcomes | Low risk | Mentioned blinding of interviewers and participants (p 3, Participants and Recruitment section). |
| Blinding of outcome assessment (detection bias) | Low risk | No blinding of outcomes but ~~used~~ the use of standardized wording with pre-coded responses and measured objectively. |
| Incomplete outcome data (attrition bias) | Low risk | No incomplete outcome data and attrition is very low. |
| Selective reporting (reporting bias) | Unclear risk | The study did not mention any specific details or indications of selective reporting. |
| Other bias | Low risk | No other potential bias observed. |

| **Taylor 2013** | | |
| --- | --- | --- |
| Methods | Randomized to receive either a print- or web-based  decision aid~~s~~, or usual care | |
| Participants | 628 + 625 + 626 male outpatients considering Prostate cancer (PCa)  screening | |
| Interventions | Intervention: web-based DA  Comparators: print-based DA and usual care | |
| Outcomes | Prostate cancer knowledge, decisional conflict, decisional satisfaction, and whether participants underwent PCa screening | |
| Notes | **-** | |
| **Risk of Bias** | **Author’s judgement** | **Support for judgement** |
| Random sequence generation (selection bias) | Low risk | Mentioned the use of computer-generated random sequence allocation to assign participants in either of the three interventions (p 3, Procedure section) |
| Allocation concealment  (selection bias) | Unclear risk | No sufficient information provided to make a judgement |
| Blinding of participants and  personnel (performance bias)  All outcomes | Unclear risk | No sufficient information provided to make a judgement |
| Blinding of outcome assessment (detection bias) | Low risk | Did not mention any blinding of outcome assessment but outcomes were measured objectively. |
| Incomplete outcome data  (attrition bias) | Low risk | Low missing outcome data and attrition rate. |
| Selective reporting (reporting bias) | Unclear risk | The study did not mention any specific details or indications of selective reporting. |
| Other bias | Low risk | No other  potential bias observed |

| **Volk 2020** | | |
| --- | --- | --- |
| Methods | Randomized to receive either a patient decision aid or standard educational material | |
| Participants | 259+ 257 current and former smokers (mostly vulnerable populations) considering lung cancer screening | |
| Interventions | DA: video- or DVD-based decision aids entitled, “Lung cancer screening: Is it Right for Me?”  Comparator: Standard educational material from a lung cancer advocacy group | |
| Outcomes | Preparedness to make a decision, decisional conflict, and knowledge of lung cancer screening | |
| Notes | - | |
| **Risk of Bias** | **Author’s judgement** | **Support for judgement** |
| Random sequence generation (selection bias) | Low risk | Mentioned the use of S-plus version 8.04 statistical software to generate a randomization schedule with different block sizes (Randomization and intervention section). |
| Allocation concealment  (selection bias) | Unclear risk | No sufficient information provided to make a judgement |
| Blinding of participants and  personnel (performance bias)  All outcomes | Low risk | No blinding of participants with regards to the intervention received but study interviewers were blinded during the 3^rd^ and 6^th^ months follow-up as questions about the PtDA were asked to the participants. |
| Blinding of outcome assessment (detection bias) | Low risk | No blinding of outcome but were measured objectively using validated questionnaires. |
| Incomplete outcome data  (attrition bias) | Low risk | Missing outcome data were minimal as well as attrition rate for both arms. |
| Selective reporting (reporting bias) | Low risk | Protocol was clearly reported in ClinicalTrials.gov. |
| Other bias | Low risk | No other potential bias was observed. |

| **Williams 2013** | | |
| --- | --- | --- |
| Methods | Randomized to receive either a decision aid or usual care | |
| Participants | 226 + 223 men considering prostate cancer screening | |
| Interventions | Intervention: decision aid booklet (home vs. clinic)  Comparator: Usual care (home vs. clinic) | |
| Outcomes | Decisional conflict, Knowledge of PCa, Satisfaction with Decision, PCa screening uptake | |
| Notes | - | |
| **Risk of Bias** | **Author’s judgement** | **Support for judgement** |
| Random sequence generation (selection bias) | Low risk | Mentioned the use of block randomization stratified by race within each site. |
| Allocation concealment  (selection bias) | Unclear risk | No sufficient information provided to make a judgement |
| Blinding of participants and  personnel (performance bias)  All outcomes | Unclear risk | No information provided |
| Blinding of outcome assessment (detection bias) | Low risk | Reported no blinding but outcomes were self-reported and were measured objectively such as use of validated questionnaires/ scales. |
| Incomplete outcome data  (attrition bias) | Low risk | No incomplete outcome data and low attrition rate |
| Selective reporting (reporting bias) | Low risk | Presented clear description of the study protocol. |
| Other bias | Low risk | No other potential bias observed |

**C. RISK OF BIAS ASSESSMENT FOR NON-RANDOMIZED STUDY DESIGNS** [ordered by study ID]

**Figure S10.** Risk of Bias Summary for Included Non-Randomized Studies

**
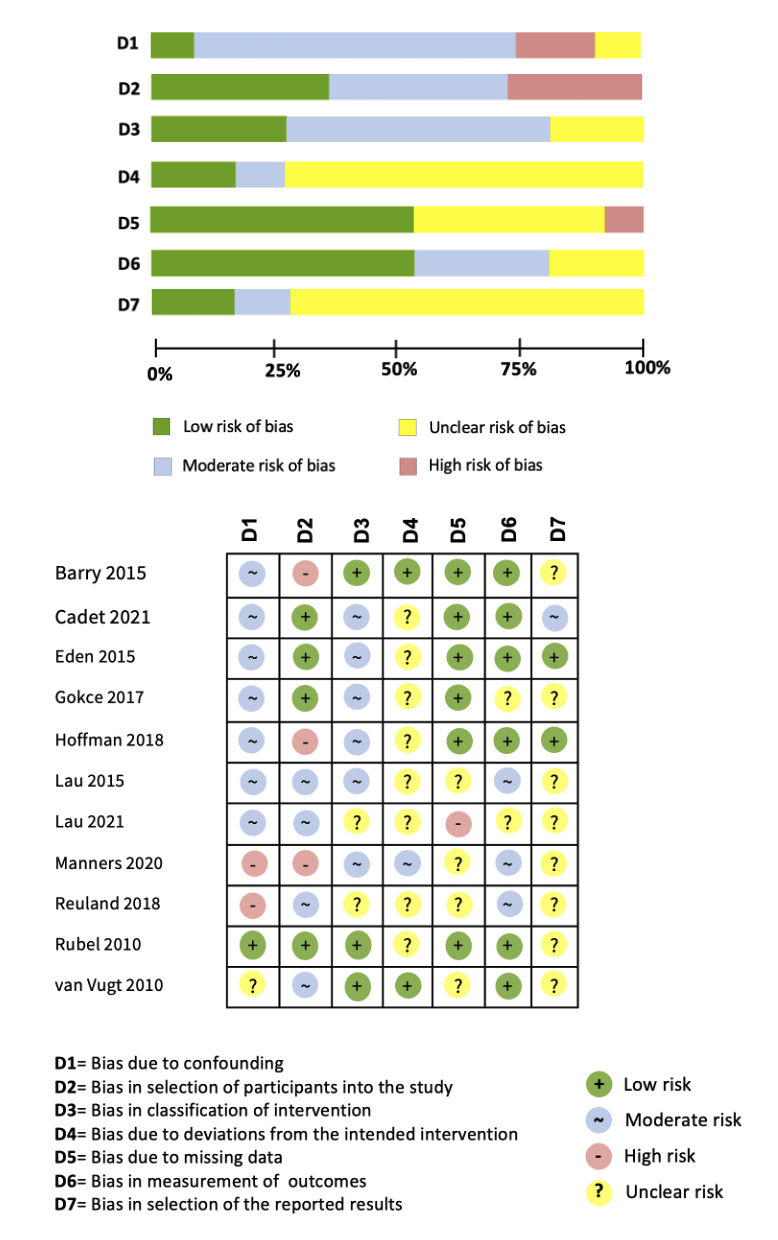
**

| **Barry 2015** | | |
| --- | --- | --- |
| Methods | Before-after study design | |
| Participants | 1,041 patients who are considering prostate cancer screening | |
| Interventions | “The PSA Test: Is It Right For You?” decision aid | |
| Outcomes captured in the review | Knowledge, leaning towards screening, their readiness to decide, and preferred role in the decision-making | |
| Notes | - | |
| **Risk of Bias** | **Author’s judgement** | **Support for judgement** |
| Bias due to confounding | Moderate risk | Although the “before” period serves as the control for the intervention group itself, it is not sufficient to address the confounding bias. |
| Bias in selection of participants into the study | High risk | The majority of the participants are highly educated, and white. Participants may not be a sufficient representation of the general population. |
| Bias in classification of intervention | Low risk | No bias with regards to classification of the intervention was observed considering that it is a before-after study design. |
| Bias due to deviations from intended interventions | Low risk | Unable to judge such deviation as there is only one intervention introduced. Also no deviations might be observed since adherence to intervention were observed. |
| Bias due to missing data | Low risk | No missing data was observed. |
| Bias in measurement of outcomes | Low risk | The researchers used validated questionnaires to measure the outcomes. |
| Bias in selection of the reported results | Unclear risk | The study did not present any protocol. Thus, there is no explicit information sufficient to make a judgement. |

| **Cadet 2021** | | |
| --- | --- | --- |
| Methods | Before-after study design | |
| Participants | 43 women aged 75 to 89 years, who are at risk for low health literacy and had a response indicating a lack of confidence in filling out medical forms | |
| Interventions | Mammography screening decision aid | |
| Outcomes captured in the review | Knowledge about mammography, decision conflict, decision-making role, acceptability, and chart abstraction | |
| Notes | - | |
| **Risk of Bias** | **Author’s judgement** | **Support for judgement** |
| Bias due to confounding | Moderate risk | The "before" period in a before-after study design acted as a control for the intervention group itself, but additional methods are needed to address confounding bias. |
| Bias in selection of participants into the study | Low risk | Follow-up and intervention synchronized; DA administered before appointments, outcomes measured after consultations (page 4, 2.2 Participants section). |
| Bias in classification of intervention | Moderate risk | The study is sound for a non-randomized study as intervention is well-defined, but not comparable to a randomized trial due to the absence of a control group. |
| Bias due to deviations from intended interventions | Unclear risk | Unable to judge such deviation as there is only one intervention introduced. Also no deviations might be observed since adherence to intervention were observed. |
| Bias due to missing data | Low risk | No missing data were present in this study as all participants were measured pre-and post-intervention and completed all questions (Page 13, Figure 1 and Table 3). |
| Bias in measurement of outcomes | Low risk | Researchers used standardized questionnaires, which are validated. |
| Bias in selection of the reported results | Moderate risk | The outcome measurements and analyses were consistent but a priori assumptions were unclear. However, outcome measures were well-defined and there were no indication of selection bias in the presented results. |

| **Eden 2015** | | |
| --- | --- | --- |
| Methods | Before-after study | |
| Participants | 75 women aged 40 to 49 years without known risk factors for breast cancer and no mammography in the previous years | |
| Interventions | Mobile application “mammopad” DA | |
| Outcomes captured in the review | Decisional conflict and intention to screen | |
| Notes | - | |
| **Risk of Bias** | **Author’s judgement** | **Support for judgement** |
| Bias due to confounding | Moderate risk | The "before" period in a before-after study design acted as a control for the intervention group itself. However, it is not sufficient to address confounding bias. |
| Bias in selection of participants into the study | Low risk | The study had minimal selection bias with high participation rates and synchronized baseline and intervention start. |
| Bias in classification of intervention | Moderate risk | The non-randomized study design is robust with a well-defined intervention, but lacks a control group for direct comparison to a randomized trial. |
| Bias due to deviations from intended interventions | Unclear risk | Unable to judge such deviation as there is only one intervention introduced. |
| Bias due to missing data | Low risk | Missing data were very low (less than 2%) (Page 1017, Table 1 and Table 3). |
| Bias in measurement of outcomes | Low risk | Questionnaires used to measure outcomes were validated and reliable tools (Page 1015, Questions and Outcome measures section). |
| Bias in selection of the reported results | Low risk | Clinic protocols were in sync with the study report and no selective reporting was observed (Page 1015, Clinic Protocol section). |

| **Gokce 2017** | | |
| --- | --- | --- |
| Methods | Before-after study | |
| Participants | men aged 40-70 years considering prostate cancer screening | |
| Interventions | American cancer society decision aid given as a PowerPoint presentation | |
| Outcomes captured in the review | knowledge, and decision conflict | |
| Notes | Did not measure decisional conflict before exposure to DA, thus, this study was not included in the meta-analysis as it only presented descriptive findings about this outcome. | |
| **Risk of Bias** | **Author’s judgement** | **Support for judgement** |
| Bias due to confounding | Moderate risk | The "before" period in a before-after study design acted as a control for the intervention group itself. However, it is not sufficient to address confounding bias. |
| Bias in selection of participants into the study | Low risk | Selection bias could be observed due to uncertainty of whether men had an anticipated 10-year life expectancy. However, measures were done to reduce this bias by reporting comorbid conditions among study populations. |
| Bias in classification of intervention | Moderate risk | Bias was observed due to implementation of intervention using PowerPoint presentation. The author presented that they might have introduced bias related to presenting the material itself. |
| Bias due to deviations from intended interventions | Unclear risk | Unable to judge such deviation as there is only one intervention introduced. |
| Bias due to missing data | Low risk | No missing data for knowledge outcomes. |
| Bias in measurement of outcomes | Unclear risk | Questionnaires used to measure outcomes were unclear. There is no sufficient information to make judgement. |
| Bias in selection of the reported results | Unclear risk | The outcome measurements and analyses were consistent but a priori assumptions were unclear. |

| **Hoffman 2018** | | |
| --- | --- | --- |
| Methods | Before-after study | |
| Participants | 30 English speaking men aged 55 to 80 years with no history of lung cancer and who are current smokers within the past 15 years. | |
| Interventions | Patient decision aid video titled "Lung Cancer Screening: Is It Right for Me?" | |
| Outcomes captured in the review | Decision-making values, screening intentions, objective knowledge, subjective ratings, values clarity, preparation for decision-making | |
| Notes | - | |
| **Risk of Bias** | **Author’s judgement** | **Support for judgement** |
| Bias due to confounding | Moderate risk | The "before" period in a before-after study design acted as a control for the intervention group itself. However, it is not sufficient to address confounding bias. |
| Bias in selection of participants into the study | High risk | Selection bias is possible due to low response rates (25% and 29%) and predominantly white, well-educated participants in the survey. |
| Bias in classification of intervention | Moderate risk | The study has a well-defined intervention but lacks a control group, limiting comparability to a randomized trial. Additionally, not all eligible patients received the intervention as it was not mandatory at selected sites. |
| Bias due to deviations from intended interventions | Unclear risk | Unable to judge such deviation as there is only one intervention introduced. |
| Bias due to missing data | Low risk | Missing data for knowledge outcomes were very low (less than 10%) (Page 523, Table 2). |
| Bias in measurement of outcomes | Low risk | Questionnaires used to measure outcomes were validated and reliable tools (Page 521, Methods section). |
| Bias in selection of the reported results | Low risk | The outcome measurements and analyses were clearly defined but a priori assumptions were unclear. |

| **Lau 2015** | | |
| --- | --- | --- |
| Methods | Uncontrolled, before-after study design recruited using convenience sampling method | |
| Participants | 60 participants who were current or former smokers and aged 45 to 80 years, had no previous history of lung cancer and had not undergone a chest computed tomographic scan in the previous year | |
| Interventions | Decision aid for lung cancer screening ([www.shouldiscreen.com](http://www.shouldiscreen.com)) | |
| Outcomes captured in the review | Knowledge, decisional conflict, concordance, and risk perception | |
| Notes | - | |
| **Risk of Bias** | **Author’s judgement** | **Support for judgement** |
| Bias due to confounding | Moderate risk | Since this study is an uncontrolled, before-and-after study, there is no control group or random assignment of participants to different interventions. Therefore, there is a possibility that participants may have received additional interventions or information outside of the study, which could confound the results. |
| Bias in selection of participants into the study | Moderate risk | Convenient sampling was used and were recruited from a specific program, the Tabacco Treatment Program. This may introduce selection bias as participants who responded may have a health-seeking behavior, mostly highly educated, and may not be a representative of the broader population, particularly people with low literacy levels. |
| Bias in classification of intervention | Moderate risk | The study has a well-defined intervention but lacks a control group. The pre-testing may have also sensitize participants about various aspects of lung cancer screening. |
| Bias due to deviations from intended interventions | Unclear risk | Participants may have received other information aside from the DA. Moreover, it is not clear whether participants were fully engaged with or follow the DA as intended. |
| Bias due to missing data | Unclear risk | The study did not mention the presence or handling of missing data. Thus, there is no sufficient information to make a judgement. |
| Bias in measurement of outcomes | Moderate risk | The authors did not mention the use of validated or reliable questionnaires to assess the outcomes. However, it was mentioned in the report that the questionnaires were adapted from the Ottawa Decision Support Framework. |
| Bias in selection of the reported results | Unclear risk | No sufficient information to make a judgement. |

| **Lau 2021** | | |
| --- | --- | --- |
| Methods | Before-after study design using a convenience sampling method | |
| Participants | 74 participants who are current or former smokers aged 45 to 77 years, without history of lung cancer, and not previously tested with the tool. | |
| Interventions | Modified web-based decision aid called shouldiscreen.com | |
| Outcomes captured in the review | Knowledge of lung cancer and screening, decisional conflict, and acceptability of the DA | |
| Notes | - | |
| **Risk of Bias** | **Author’s judgement** | **Support for judgement** |
| Bias due to confounding | Moderate risk | Confounding may arise from uncontrolled factors that could influence the outcome apart from the intervention. Although the before period serves as a control group, additional methods are needed to address confounding bias.. |
| Bias in selection of participants into the study | Moderate risk | Potential bias in the selection of participants could be observed due to the use of convenience sampling approach. However, the study appears to have some diversity, as it includes participants from African American community in Detroit. |
| Bias in classification of intervention | Unclear risk | No sufficient information to make a judgement. |
| Bias due to deviations from intended interventions | Unclear risk | It is possible that deviations from the intended intervention may occur in this study due to confounding, however there is no sufficient information to make a judgement with regards to this deviations or on how authors minimized such deviations. |
| Bias due to missing data | High risk | There were missing responses and misconception in the value clarification portion, indicating limitations in participants’ ability to complete the exercise independently. |
| Bias in measurement of outcomes | Unclear risk | The authors mentioned that the questionnaires had good psychometric properties. However, it is unclear whether questionnaires were validated and reliable enough to assess the outcomes. |
| Bias in selection of the reported results | Unclear risk | No sufficient information to make a judgement regarding the selective reporting of this study. |

| **Manners 2020** | | |
| --- | --- | --- |
| Methods | Quasi-experimental pre- and post-exposure study design | |
| Participants | 342 individuals aged 55 to 79 years, who are current or former smokers. | |
| Interventions | Two-stage resource for lung cancer screening including two pamphlets: a tri-recruitment pamphlet for screening eligible individuals and a consumer education pamphlet for screening-ineligible individuals (**the PtDa and ineligible pamphlets**). | |
| Outcomes captured in the review | Lung cancer eligibility, decisional conflict, knowledge about screening, satisfaction with the pamphlets, and screening preferences. | |
| Notes | - | |
| **Risk of Bias** | **Author’s judgement** | **Support for judgement** |
| Bias due to confounding | High risk | The study included pre-post survey of eligible and ineligible cohorts. However, the cohorts have imbalanced baseline characteristics and no statistical adjustments were done to account for confounders. |
| Bias in selection of participants into the study | High risk | Potential selection bias in this study includes the setting being a secondary hospital, missing smoking history data, and nonparticipants being more likely to be current smokers, which limits the generalizability of the findings (Discussion, page 5). |
| Bias in classification of intervention | Moderate risk | Possible bias introduced by the researcher verbally informing screening-ineligible participants about their ineligibility, which may influence their responses and decision-making. Lack of formal assessment of delivery fidelity for both PtDA and ineligible pamphlets is also observed. |
| Bias due to deviations from intended interventions | Moderate risk | Variations in the delivery and presentation of the pamphlets to participants could occur as well as the potential differences in understanding, interpretation, or adherence to the intended content and messaging of the pamphlets. |
| Bias due to missing data | Unclear risk | The study did not mention how the researchers handled missing data nor discussed specific strategies to mitigate the biases arising from missing data. |
| Bias in measurement of outcomes | Moderate risk | The authors mentioned that they did not use validated questionnaires to assess the outcomes of this study. However, such questionnaire was adapted from the predated publication of a recent validated lung cancer screening knowledge questionnaire. |
| Bias in selection of the reported results | Unclear risk | No sufficient information to make a judgement. |

| **Reuland 2018** | | |
| --- | --- | --- |
| Methods | Pre-post design with baseline and follow-up surveys | |
| Participants | 50 primary care patients aged 55 to 80 who are current or former smokers. | |
| Interventions | Video decision aid on lung cancer screening | |
| Outcomes captured in the review | Knowledge of the benefits and harms of screening, screening preferences, screening behavior within 3 months of viewing the decision aid, relationships between screening knowledge, preferences, and screening test ordering during subsequent primary care encounters. | |
| Notes | - | |
| **Risk of Bias** | **Author’s judgement** | **Support for judgement** |
| Bias due to confounding | High risk | Lack of control group makes it difficult to establish causality. Other factors may influence screening behavior and preferences. |
| Bias in selection of participants into the study | Moderate risk | Primary providers had the discretion to exclude patients based on comorbidities, introducing potential selection bias. |
| Bias in classification of intervention | Unclear risk | No sufficient information to make a judgement. |
| Bias due to deviations from intended interventions | Unclear risk | No sufficient information to make a judgement. |
| Bias due to missing data | Unclear risk | 10 participants who participated in pre-test phase were exclude. However, it was unclear with regards to the reason for exclusion of these individuals. |
| Bias in measurement of outcomes | Moderate risk | Knowledge assessment may be subjective. Preferences and behaviors may be influenced by social desirability bias. |
| Bias in selection of the reported results | Unclear risk | Selective reporting of results may introduce bias in favor of positive findings. Other significant findings or negative results may not be reported. However, there is no protocol that was mentioned in the study and thus, it is difficult to make sufficient judgement. |

| **Rubel 2010** | | |
| --- | --- | --- |
| Methods | Solomon four-group design with (1) pretest, decision aid, immediate posttest; (2) pretest, no decision aid, immediate posttest; (3) no pretest, decision aid, immediate posttest; and (4) no pretest, no decision aid, posttest only. | |
| Participants | 200 male non-Hispanic white participants recruited from the general population | |
| Interventions | Pretest instrument and decision aid  Condition 2 group: Pretest, no decision aid, posttest  Condition 4 group: No pretest, no decision aid, posttest only | |
| Outcomes captured in the review | Decisional anxiety, decisional conflict, decision control preferences, knowledge, perception of risk and PSA screening schema | |
| Notes | African American men were excluded from the study due to a separate evaluation of a specific decision aid for prostate cancer screening | |
| **Risk of Bias** | **Author’s judgement** | **Support for judgement** |
| Bias due to confounding | Low risk | The presence of confounding factor was addressed by the use of Solomon four-group approach. Study findings show no significant demographic differences among participants in each group. |
| Bias in selection of participants into the study | Low risk | Study participants were mostly non-Hispanic whites. However, the author clearly stated that this was due to a separate analysis done for African-American participants. |
| Bias in classification of intervention | Low risk | The information provided in the report does not indicate any specific bias in the classification of the intervention. |
| Bias due to deviations from intended interventions | Unclear risk | No sufficient information provided in the report to make a judgement. |
| Bias due to missing data | Low risk | No missing data was observed. |
| Bias in measurement of outcomes | Low risk | The use of validated self-administered, paper-and-pencil instruments for outcome measures addressed potential measurement errors in assessing the outcomes. |
| Bias in selection of the reported results | Unclear risk | No sufficient information provided in the report to make a judgement. |

| **van Vugt 2010** | | |
| --- | --- | --- |
| Methods | Intervention study | |
| Participants | 2000 men who were randomly sampled, aged 55 to 65 years from the population registry of the city of Dordrecht, the Netherlands | |
| Interventions | Leaflets about Prostate cancer (PSA) and the benefits of screening as well as a risk indicator (PRI) | |
| Outcomes captured in the review | Informed choice, including knowledge, attitude towards PSA testing, and intention to have a PSA test, anxiety, prostate cancer anxiety, health-related quality of life and decisional conflict | |
| Notes | - | |
| **Risk of Bias** | **Author’s judgement** | **Support for judgement** |
| Bias due to confounding | Unclear risk | The presence of confounding variables is not mentioned in the report, so it is unclear how the authors addressed this potential bias. |
| Bias in selection of participants into the study | Moderate risk | Men who completed both questionnaires were compared to those who only completed questionnaire 1. Potential selection bias may occur due to the exclusion of participants who were not able to complete questionnaire 1. |
| Bias in classification of intervention | Low risk | Authors mentioned that the leaflet was tested with a target population and found to provide balanced and accurate information. |
| Bias due to deviations from intended interventions | Low risk | The information provided does not indicate any significant deviation from the intended interventions. |
| Bias due to missing data | Unclear risk | Attrition rate (those who did not complete questionnaire 2) was about 17.6%. However, there was no mention regarding how missing data were handled. |
| Bias in measurement of outcomes | Low risk | The authors used validated questionnaires and scales to measure various outcomes, including knowledge, attitude, intention, and psychological measures. |
| Bias in selection of the reported results | Unclear risk | No sufficient information to make a judgement. |
