# Supplementary material for: Mixed-Method Systematic Review and Meta-Analysis of Shared Decision-Making Tools for Cancer Screening
Source: Cancers (Basel). 2023 Jul 29;15(15):3867. doi: 10.3390/cancers15153867 (PMC10417450; doi:10.3390/cancers15153867)
Supplement: Supplementary file 1 [file cancers-15-03867-s001.zip › cancers-2455744-supplementary/Supplementary file_2_Summary characteristics copy.docx]

| **Table S1.** Characteristics of the shared decision-making tools for vulnerable populations | | | | | | | |
| --- | --- | --- | --- | --- | --- | --- | --- |
| **Study ID** | **Framework/criteria/guideline used to develop the SDM tool** | **Format of the SDM tool** | **Content of the SDM tool** | **Readability** | **Length of time in using the SDM tool** | **When it is delivered** | **Medical screening decision** |
| Allen 2010 ^1^ | Ottawa Decision Support Framework and IPDAS criteria | Computer-based DA***** | (1) Prevalence of Prostate cancer screening (CaP), (2) CaP risk factors, (3) methods used for early detection and their operating characteristics (i.e., sensitivity, specificity), (4) potential advantages and disadvantages of screening, and men's evaluation of the relative importance of these factors, (5) recommendation of major medical organisations that men make individualised decisions, (6) meaning of an elevated PSA test, and (7) methods for diagnosis. | 6^th^ grade reading level | 28-min. | Before consultation | Prostate cancer screening (CaP) |
| Brenner 2016 ^2^ | Not reported | Web-based DA, written in Flash CS4 professional***** | (1) introduction and review of faecal testing (FOBT/FIT) and colonoscopy, (2) direct comparison of the features of the two testing, | ≤ 8^th^ grade reading level ^a^ | 14-min. | Before consultation | Colorectal cancer |
| Cadet 2021 ^3^ | Based on IPDAS criteria and Ottawa Decision Support Framework | Paper-based DA***** | (1) information on older women’s breast cancer risk, (2) LE, (3) outcomes of screening, (4) competing mortality risks, (5) breast cancer treatments, and (6) a value clarification exercise. | Not reported | Not reported | Before consultation | Breast cancer |
| Gokce 2017 ^4^ | Developed by ACS staff and volunteers in conjunction with the publication of the 2010 ACS guideline for prostate cancer early detection | PowerPoint presentation***** | (1) Prostate cancer statistics, (2) detailed key information of on the potential benefits and harms of PSA screening | 5^th^ grade reading level | Not reported | Before consultation | Prostate cancer |
| Hoffman 2017 ^5^ | Based on Ottawa Decision Support Framework, the Integrated Model of Behaviour, and the Edutainment Decision Aid Model | Video-based patient DA presented in an entertainment-education format ***** | (1) anatomy of digestive system and colon, (2) how CRC forms, (3) who is at high risk of developing it, (4) morbidity/ mortality rates (5) benefits of early detection (6) detailed description of and comparison screening options, (7) preparations required by the patient, (8) other pros and cons, (9) statements encouraging patients to communicate to their provider about CCS. | Not reported | 21.67-min. | Prior to their next clinic visit | Colorectal cancer |
| Hoffman 2018 ^6^ | Based on IPDAS criteria and on theories in cognitive psychology and decision making, including the integrative model of behaviour | Video-based DA ***** | (1) risk of radiation exposure by comparison with other medical imaging and environmental exposure, (2) false positives using the term false alarms and (3) depicts the false positive rate using an icon array, (4) risk of overdiagnosis, (5) value clarification, (6) summary of potential benefits and harms, and (7) the importance of considering personal decision with their doctor. | Not reported | Not reported | Before consultation | Lung Cancer |
| Housten 2020 ^7^ | Not reported | 3 formats: (1) an audio booklet, (2) a video with static images and (3) a video with animated images (final DA) | (1) briefly described colon anatomy, polyps, CRC, and CRC screening, (2) recommendations for regular screening, (3) CRC screening tests, (4) description of each test procedure, (5) its advantages, (6) an explanation of test results, (7) recommended test frequencies, (8) what to expect before, during, and after each test, (9) CRC risk information. | 8^th^ grade reading level | 3.51-min video | Before consultation | Colorectal cancer |
| Lau 2021 ^8^ | Based on IPDAS criteria, the Centers for Medicare, and Medicaid Services (CMS), and USPSTF | Web-based DA***** | (1) Basic information about low dose computed tomography screening, (2) education on lung cancer risk calculator that computes a personalised risk based on PLCOm2012 model, (3) information about insurance coverage (additional). | Not reported | 5 to 10 minutes | Before consultation | Lung cancer |
| Lepore 2012 ^9^ | The intervention was based on a combination of prior studies, practice guidelines, and decision support theory with an emphasis on education, values clarification, and SDM | Educational pamphlet called “Prostate Cancer: Your Life-You Decide” + Tailored telephone education calls***** | Pamphlet included (1) advantages and disadvantages of prostate cancer testing, (2) risk factors, and (3) potential risks and benefits of testing.  Telephone call intervention focused on (1) establishing rapport, (2) providing balanced information, (3) helping men clarify testing preferences, and (4) preparing for discussions with physicians. | Not reported but identified to be designed for men with low literacy levels | 20 minutes with SD= 2.3 (initial call) | Before consultation | Prostate cancer testing |
| Manners 2020 ^10^ | Based on IPDAS criteria | Eligible pamphlet | (1) descriptions of lung cancer screening process, (2) screening benefits, (3) potential harms, (4) available choices, (5) value clarification exercise. | Not reported | Not reported | Before consultation | Lung cancer |
| Miller 2011 ^11^ | Not reported | One-page colour handout***** | (1) a short introductory overview of CRC screening including CRC prevalence, (2) the rationale for screening, and (3) a description of common screening tests (FOBT, flexible sigmoidoscopy, and colonoscopy). | ≤  8^th^ grade reading level | 10.1-min. | During consultation*** | Colorectal cancer |
| Miller 2018 ^12^ | Not reported | iPad app mPATH-CRC Program***** | (1) Overview of CRC screening, (2) description of screening tests, (3) benefits of CRC screening, (4) risks and limitations associated with CRC screening tests, (5) personalized screening options, and (6) follow-up electronic messages. | Not reported | 8.6 minutes | Before consultation | Colorectal cancer |
| Perestelo-Perez  2019 ^13^ | Not reported | Web-based DA  (Also available in printed format or via email)***** | It is organised in three sections; (1a) explains the usefulness of DAs and (1b) a summary of their content. (2b) explains CRC causes, (2b) symptoms & (2c) available treatments. (3) information on FOBT and colonoscopy is presented, including quantitative data about (3a) incidence, (3b) mortality risk reduction, and (3c) potential adverse effects. | Not reported | Not reported | During consultation*** | Colorectal cancer |
| Ruparel 2019 ^14^ | Based on USPSTF | Information film + information booklet***** | Both interventions discussed (2) lung cancer, (2) the benefits and harms of LCS (including indeterminate pulmonary nodules and false positives, overdiagnosis, and radiation damage), (3) the LDCT procedure, and (4) the possible results after the scan. | Not reported | 5 to 10-min. | Not reported | Lung cancer |
| Rubel 2010 ^15^ | Not reported | 17-page booklet***** | The booklet provides the following: (1) basic information about the prostate gland and prostate cancer, (2) a description of prostate cancer screening, (3) a description of varying opinions of medical experts about prostate cancer screening, and (4) a description of potential follow-up testing and treatment for prostate cancer. | grade 8.4 reading level | 11.58 min. | Not reported | Prostate cancer |
| Salkeld 2016 ^16^ | Annalisa is based on Multi Criteria Decision Analysis (MCDA) | A software -based prescriptive decision aid***** | The software included (1) questions about the relative importance of attributes (benefits and harms) related to the options, (2) pre-populated information, expressed as probabilities of outcomes, (3) evidence for screening, and (4) generated overall opinion based on selected options. | Not reported | 18 to 22-min. | Not reported | Prostate cancer |
| Schapira 2019 ^17^ | Based on the framework developed by Shaffer and colleagues (cite.) | Web-based DA***** | (1) ascertainment of breast cancer risk factors needed for the National Cancer Institute Breast Cancer Risk Assessment Tool (NCI-BCRAT), (2) an introduction to the decision problem including (2a) description of different guidelines, (2b) risks, and benefits of screening, and (2c) decision aid goal), (3) a table outlining USPSTF and ACS guidelines, (4) comparison of mortality reduction attributed to mammography by age group, (5) pictographs depicting outcomes of mammography, (6) and 10-year and lifetime risk. | Not reported | 10.2 min. | After consultation | Breast cancer |
| Schroy 2011 ^18^ | Based on Ottawa Decision Support Framework, ACS, and USPSTF recommendations | Web-based DA***** | (1) importance of screening, (2) intended purpose of the tool and instructions in its use; (3) an overview of the epidemiology of CRC, (4) natural history, (5) rationale for screening, (6) benefits of screening, (7) the availability of multiple screening options, and (8) the lack of consensus regarding a best screening method, and (9) brief descriptions of the all five recommended screening methods. | Not reported | Not reported | During consultation | Colorectal cancer |
| Schwartz 2019 ^19^ | Based on IPDAS criteria | Quantitative and verbal DA | (1) information regarding CRC, (2) comparative effectiveness of colonoscopy and FIT (presented as frequencies depicted on icon charts), including (2a) sensitivity of a single application of colonoscopy and FIT, (2b) average lifetime CRC incidence and mortality with or without CRC screening, (2c) frequency of single FIT turning positive, and (2d) frequency of complication from a colonoscopy. | Not reported | 4.5 min video | Not reported | Colorectal cancer |
| Sferra 2021 ^20^ | Not reported | 1-page Information sheet ***** | Not reported***** | 6^th^ grade reading level | Not reported | During consultation | Lung cancer |
| Smith 2010 ^21^ | Based on IPDAS criteria | Paper-based booklet or video- based PtDA***** | The booklet provides the following: (1) basic information about the prostate gland and prostate cancer, (2) a description of prostate cancer screening, (3) a description of varying opinions of medical experts about prostate cancer screening, (4) a description of potential follow-up testing and treatment for prostate cancer, and (5) an interactive exercise to identify their risk of bowel cancer and to clarify their values. ***** | 7^th^ grade reading level | Not reported | Before consultation | Bowel cancer |
| Volk 2020 ^22^ | Based on IPDAS criteria and USPSTF | Video- or DVD-based PtDA***** | (1) eligibility for lung cancer screening, (2) a calculation of tobacco pack-year smoking history, (3) lung cancer epidemiology and risk factors, (4) a video of a patient in CT scanner, (5) icon array to graphically depict the magnitude of mortality reduction, false positive results, and harms from invasive procedures, and (6) other radiation exposure, including screening mammography. ***** | Not reported | 9.5 min | Before consultation | Lung cancer screening |
| Williams 2013 ^23^ | Based on CDC PCS educational tool | Printed-based DA in booklet form***** | (1) information on the leading causes of death among men, (2)  the accuracy of the PSa test, (3) PCS guidelines, (4) PCa diagnostic procedures and treatments, and (5) the 10-item values clarification section. ***** | 8^th^ grade reading level | 20 min. | During consultation | Prostate cancer |

| **Table S2.** Characteristics of the shared decision-making tools reported in each study for non-vulnerable populations | | | | | | | |
| --- | --- | --- | --- | --- | --- | --- | --- |
| **Study ID** | **Framework/criteria/guideline used to develop the SDM tool** | **Format of the SDM tool** | **Content of the SDM tool** | **Readability** | **length of time**  **in using the**  **SDM tool** | **When it is delivered** | **Medical Screening Decision** |
| Barry 2015 ^24^ | Based on IPDAS criteria | DVD, booklet, or online DA | (1) Options of deciding to have or not to have PSA screening, (2) pros and cons of each choice | Not reported | 31 min. (video portion) | After consultation | Prostate-specific antigen |
| Eden 2015 ^25^ | Based on IPDAS criteria | Web-based application | (1) breast cancer risk assessment using the same questions as the Breast Cancer Surveillance Tool (BRST) instrument, (2) information modules including breast cancer, mammography, and the screening experience, (3) priority setting activity, (4) customized report including user’s screening priorities including benefits and harms of breast cancer screening, intentions for screening, concerns, and questions. | 8^th^ grade reading level | Not reported | Before consultation | Breast cancer screening with mammography |
| Halley 2015 ^26^ | Not reported | DVD DESI +booklet or Web-based DESIs***** | (1) Multiple screening options, (2) risks and benefits of each option, including colonoscopy, faecal occult blood test (FOBT), sigmoidoscopy, and CT colonography | Not reported | 39 min. | During consultation | Colorectal cancer |
| Lau 2015 ^27^ | IPDAS version 4.0 checklist, USPSTF* guidelines, and current risk communication best practices | Web-based DA***** | (1) Personalized LC risk calculator, (2) risk factors, (3) harms and benefits of LDCT screening, (4) an explicit values clarification exercise | Not reported | 10 min. | Not reported | Lung cancer |
| Lewis 2015 ^28^ | Not reported | DVD format***** | (1) A description prostate cancer screening/tests, (2) treatment options if cancer is detected, (3) associated benefits and risks of treatment options, and (4) uncertainty of mortality benefit of treatment | Not reported | 31 min. | Before consultation | Prostate-specific antigen |
| Lewis 2018 ^29^ | Based on IPDAS criteria and the Ottawa Decision Support Framework | paper-based tool***** | (1) A description of FOBT, (2) a description of screening colonoscopy and its potential harms, (3) information about positive stool tests requiring follow-up diagnostic colonoscopy, (4) a description of the importance of competing mortality with targeted information based on age and gender, (5) why individualised decision making is necessary for older adults, (6) the need to weigh the harms and benefits of CRC screening, and (7) a values-clarification exercise. | 7^th^ grade reading level | 5 to 15 min. | during consultation or visit | Colon cancer |
| Reuland 2018 ^30^ | Estimates were based on NLST trial data | Video-based DA***** | (1) Rationale for screening, (2) eligibility criteria, (3) a description of the LDCT procedure, and (4) a dynamic icon array (pictogram) that sequentially depicted estimates for benefits and harms of screening among 1000 individuals screened annually for 3 years. | Not reported | Not reported | during consultation or visit | Lung Cancer |
| Sepucha 2022 ^31^ | Used Ottawa Decision Support coaching techniques. ** | Printed 3-page decision worksheet + telephone session***** | The decision worksheet presented the pros and cons of three options: (1) colonoscopy, (2) stool-based test, and (3) delay screening until next year. | Language translated in Spanish | Not reported | Before consultation | Colorectal cancer |
| Sheridan 2012 ^32^ | Not reported | video-based DA + researcher-led coaching session for patients + education session for providers | (1) The prostate specific content, (2) information about the prevalence of cardiovascular disease and (3) colon cancer, (4) the certain benefit of screening for these diseases, and (5) the options and attributes of common screening tests and treatments for these diseases. | Not reported | 120 min | After consultation | Prostate cancer |
| Sheridan 2016 ^33^ | Based on USPSTF recommendation | 1-page decision support sheet | (1) Description of disease for which screening could be undertaken (e.g., disease incidence and mortality rates), (2) description of the screening test and its benefits, (3) physical and psychological harms across screening cascade, and (4) an encouragement to decide. | Written in 8^th^ grade level | Not reported | Before or after consultation | Prostate and Colorectal cancer |
| Taylor 2013 ^34^ | Based on IPDAS criteria, American Urological Association guidelines, and USPSTF | Web-based DA or Print-based DA***** | The printed DA (1a) what prostate gland is; (2a) description of screening options, risks, (3a) treatment options, risks, adverse effects, (4a)  PCa risk factors, and (5a) encouragement to discuss screening with a physician (6a) a 10-item values clarification tool, and (7) resources for more information.  The web DA includes (1b) a voice-over that presents most of the text, (2b) pop-up definitions of 77 terms, (3b) 8 video testimonials, (4b) an interactive values clarification tool, and (5) figures, animation, and graphics. | Both DAs have 8^th^ grade reading level | Web-based DA: 30-50 min. | Before consultation | Prostate cancer |
| van Vugt 2010 ^35^ | Based on the screening results of 6288 men participating in the initial screening round of the ERSPC section Rotterdam, the Netherlands | Leaflet + risk indicator***** | Leaflet PRI: (1) information about PCa, (2) the pros and cons of PCa screening, (3) a risk indicator to calculate their own estimated risk of having PCa.  Risk indicator- Level 1: (1) information on family history, (2) age, and (3) urinary function to calculate a rough estimation on the probability of having a biopsy detectable PC. | Not reported | Not reported | Before consultation | Prostate cancer |

* significant effect favouring intervention; IPDAS criteria: International Patient Decision Aid Standard criteria (<http://decisionaid.ohri.ca/AZsumm.php?ID=1148-effect>), USPSTF: US Preventive Services Task Force, ^HL^: High literacy, ^LH^: Low literacy, ^a^ SDM tool characteristics were retrieved from study protocols or other publications on the tool.

**Table S3.** Summary characteristics of included qualitative studies

| **Study ID,**  **country** | **Study design** | **Participants (n)** | **Sample size**  **(n)** | **Data analysis approach** | **Summary results** | **Sample quotations/items** |
| --- | --- | --- | --- | --- | --- | --- |
| Akanuwe 2019 ^36^, England | Semi-structured individual interviews and focus groups | service users aged 21 to 71 years, without active cancer but without symptoms suggesting cancer | 19 | Used a priori codes derived from the Risk Analysis Framework | Participants suggested personalising risk information, involving patients, openly sharing risk information, and allowing sufficient time during tool use. | *“You wouldn't want to feel that you've been rushed, you would want them to take time to talk with you, and if they try to cut this conversation short you would think that they didn't care, and again that could reduce your confidence”* (Service User 12: individual interview) |
|  |  | general practitioners and practice nurses aged 33 to 55 years (n=17) | 17 |  |  | *“If you don't tell them before using the tool it means you are not being honest. I mean you can't do anything without telling the patient, you need their consent”* (Practitioner 4 [GP]: individual interview). |
| Amélie 2022 ^37^ , France | Grounded theory using individual interviews | Women aged between 42 and 75 years | 13 | Content analysis/Thematic analysis using an inductive analysis based on grounded theory | Participants desired an intuitive, patient-centered decision aid to enhance knowledge, harmonize practice, and provide reliable information, while recognizing potential limitations. | *“It would be great to have information about breast cancer: things would be clearer for us”* (Women 3). |
|  |  | General practitioners, gynaecologists, midwives, radiologists, and screening program managers | 27 |  |  | *“It would be great to have that sort of tool. It would help to harmonise things”* (Midwife 3).  *“It has to be something visual, something integrated into software. […] It needs to be easy to access”* (GP 4). |
| Baptista 2020 ^38^, Portugal | Semi structured, individual interviews | Portuguese native-speaking men aged 55 to 69 years old without previous diagnosis of prostate cancer | 15 | Thematic analysis using a deductive and inductive content analysis approach | Five main themes are presented: informational content, information comprehension, sociocultural appropriateness, feelings and main message and personal perspective regarding PCa screening. | ‘*This was good, this information should be available to 90% of the population. (…) Society still hides a lot (…) that’s one of the reasons why it’s important to educate men to open themselves to talk about this with their doctor. (…) Some parts I found the language a little bit technical’ [B3].* |
| Croes 2019 ^39^, United States | Delphi technique | Women aged 40 to 49 years | 10 | Consensus reached through survey rounds using predefined criteria | Panellists agreed on the importance of understandable information, knowledge of personal risk factors, and effective communication by PCPs. | *“PCPs should tell women that their risk of breast cancer is based on their personal risk factors* (item that achieved consensus alighted with the theme information delivery and patient education).” |
|  |  | Primary care providers (PCPs) and healthcare decision scientists | 20 |  |  | *“Healthcare systems should train PCPs to provide clear explanations to women about the risks and benefits of mammography screening* (item that achieved consensus aligned with the theme” interpersonal clinician-patient communication). |
| Crothers 2016 ^40^, United States | Mixed-method study (use of focus group discussions) | Outpatients aged 50 to 74 years who are current or former smokers for at least 20 pack-years | 45 | Qualitative part used thematic analysis | Participants lacked awareness, desired information on benefits/harms, emphasized effective communication, found decision aids influential, and sought personalized discussions. | “…*putting it in plain English*,” and avoiding “*the*  *doctor’s language.*”  Some patients preferred the term “*false alarm*” to “*false positive,”* and reflected that the term “*positive*” could create confusion: “*Positive means different things to different people. The word positive can be misconstrued*”. |
| Dubenske 2021 ^41^, United States | Mixed-method (open-ended satisfaction survey items) | English-speaking women aged 40 to 49 years | 53 | Conventional exploratory content analysis | Patient feedback: BCARE-DA themes: ease, clear content, personalized risk; Improved physician communication. Concerns: SDM time, influenced by other factors. | Not reported |
|  |  | Physicians | 11 |  |  |  |
| Engelen 2016 ^42^, Belgium | Semi structed interviews and group discussions | Men 50 years and older eligible for ED of CaP | 43 | Thematic analysis following COREQ guidelines | Attitudes toward shared decision making were generally positive. Strategies suggested to support DA use: supplementing with short tools, training programs, and public awareness campaigns. | *It is good that [the DA] will be there – because otherwise you’re 100% dependent on what your GP will tell you. Also, on the internet you’ll only find contradictory information that doesn’t help you very much. So I think it’s very good [that it exists].* [Group discussion men 5] |
|  |  | General practitioners in Flanders, Belgium | 16 |  |  | *Giving GPs the opportunity to practice with [the DA] so that they can browse it quickly. ... For example, during LOK meetings [i.e., periodically organized meetings for physicians as part of permanent education], ... Practical training, concrete: A patient sits in front of you, this is his question, he asks for a blood test, for a PSA test, how are you going to deal with this?* [Interview GP 4] |
| Friedman 2012 ^43^, United States | Exploratory study using focus group discussions | African American men and women | 22 | Thematic analysis | Men discuss prostate cancer more with women, particularly their partners, than with healthcare providers or other men. Barriers include fear, discomfort with screenings, embarrassment/pride, and masculinity, affecting discussions among African American men. | *“They don’t talk about it because they get afraid. You*  *know, you say something like cancer, you know, people get afraid to talk about it.”*  *“He doesn’t like talking to doctors. He doesn’t like talking to family members because it’s his business and not theirs. They do not like to share that kind or any kind of stuff, actually, about themselves with family because remember, they got to be macho.”* |
| Halley 2014 ^26^, United States | Exploratory, randomized mixed-method crossover study | Participants eligible for colorectal cancer screening with mean age of 64 years | 90 | Thematic analysis | Participants preferred self-directed website for personalized information; others found DVD's comprehensive overview helpful. | *"I think the website would probably be a better first choice because again you could pick out the topics that you really wanted to find out something about and skip the ones that you knew something about"* (Male, Age 71, BPH).  *"I [want] the type of information on the website if I really want to dig in, but if I want an overview of where to start digging, where to go, where I want more information from, the DVD is far better"* (Male, Age 78, BPH). |
| Kuss 2021 ^44^, Germany | Qualitative Sequential design | Men aged 55 to 69 years  Physicians | 32  9 | Framework approach using thematic and central charts | Comprehensive information needed, addressing accuracy, benefits, consequences, interpretation support, and physician's attitude. Empowered decision-making | *“But […] for me as a patient it would be important to have*  *all this in written form and then to have a look-see myself.*  *Calmly. […] I guess, when sitting at a doctor, you will be*  *overloaded, of course. So, you sit there, you gather things*  *and then I guess, […] only at home I really begin to think*  *about it”* (0102; man)*.* |
| Hernandez-Leal 2022 ^45^, Spain | Mixed methods study using a Delphi Technique with three rounds | Healthcare professional | 20 | Thematic analysis | The participants found three-talk model suitable, expressed satisfaction with the handbook and guide, and suggested additional content. | *“Provide more information or example dialogues on how to use communication skills. This last (point) if the health professionals don’t have a grounding or training in active listening, motivational interviewing, empathy, reflection, etc”* (P3*).*  *“ Change relative risks to absolute risks”* (P15)*.* |
| Maschke 2020 ^46^, United States | Interview-based qualitative study design | Women aged 40 to 45 years old who are outpatients | 25 | Content analysis | Four themes identified including family history vs. risk assessment, potential harms, information delivery, and pre-visit tools. Counselling techniques may not meet patient needs; improvements and increased accessibility are needed. | *A pie chart, graphs, I’m more so that kind of person, but [the icon array] doesn’t do nothing for me” (Patient 5).* |
|  |  | Primary care providers | 20 |  |  | *“I don’t tend to get into those [potential harms] details because I feel like it gets us off track and then people are less likely to be willing to do it”(PCP 2).* |
| Pannebakker 2019 ^47^, United Kingdom | Qualitative in-depth semi structured interviews | Patients aged 36 to 80 years  General Practitioners | 14  14 | Thematic analysis using a coding and categorization approach | GPs found the electronic clinical decision support (eCDS) tool useful, time-efficient, and facilitating communication. However, they have concerns about the policy alignment and unnecessary referrals. | “Regarding computer use: *“Sometimes you can go there and see one and he never looks at you at all and he just types, but this one, he did sit and look at it, and then type it in, you know”* (04, Female,70 years). |
| Reese 2022 ^48^, United States | Qualitative interview-based using a theoretical model | Attending physicians | 14 | Thematic analysis | Identified key barriers and proposed implementation strategies including the use of decision aids and shared decision making. | *“I will say that, given the number of competing interests in the chart and with patients, this might be viewed by some people as just too much, because a lot of us view a lot of things as already too much. I’m just trying to be realistic.”* (Patients) |
| Schapira 2019 ^49^, United States | Qualitative structured interviews | Adult men who are dominantly black or African American | 22 | Thematic analysis using inductive approach | Patients prefer decrease uncertainty motivated screening decisions. Uncertainty influences how patients weigh screening benefits and harms. | *“I’ll put in this way: it’s the fear of the unknown. I’m really afraid of what I don’t know, okay? Some people don’t want to know because they’re afraid to find out the answer. I fear what I don’t know because what I know I feel like I can control it or I can get help—allow someone to control it, like a doctor or whatever”*  (Interview #8, male). |
| Schonberg 2019 ^50^, United States | Qualitative study with in-person individual interviews | Non-physician healthcare team members and primary care practices | 32 | Thematic analysis | Participants found the decision aid clear and balanced but suggested shortening it for low health literacy patients. Most participants recommended providing the tool to patients before visits, with clinicians’ approval and support. | *“Before a visit with their primary so they can ask questions.”*  *“Most patients will still want to have a discussion with their PCP and make a decision based on his/her opinion.”*  *“If we had one of the nurses go over it with them so that before they left they were confident in their decision.* |
| Schwartz 2021^51^, United States | Qualitative focused group discussions | Laypersons living within a 25-mile radius aged 18 and above | 28 | Content analysis | Twenty-one deliberative conclusions were strongly supported, including recommendations for disclosure of cost, simplicity and baseline risk of colon cancer. | *“I don’t think you need to have an exhaustive list of everything because that could go on for pages. But the common, the bleeding, the tears, um, certainly those top three or four things, list them, because that was part of being informed”* (Participant 9088; large group, day 2).  *“[If] you’re only 60 out of 1,000, then that doesn’t encourage you to go get a test. Uh, I think it should be that the baseline risk is minimal . . . I don’t think the data should be divulged”* (Participant 401; large group, day 3). |
| Tatari 2021 ^52^, Denmark | Qualitative study using focus group discussion and interview | Women from 10 non-Western countries aged between 27 and 59 | 37 | Thematic analysis | Women suggested information material in their own language with a simple, positive, and concrete communication strategy. They would like to be involved in an awareness strategy and share the knowledge with their network. | *“That letter doesn’t work for me- I’m missing something else like this [the short presentation]”* (Focus group 1).  *“First you should tell about screening, what it is and why it’s relevant, and then you should hand out a leaflet in their native language…and maybe some extras we can pass on”* (Focus group 3). |
| Toledo-Chávarri 2016 ^53^, Spain | Qualitative study using focus group discussion | Women aged 40 to 69 years  Healthcare professionals | 39  23 | Content analysis | Women value receiving information on benefits and harms of breast cancer screening but struggle with understanding certain concepts. Professionals highlighted the need for improvement. | *“If only one is diagnosed with cancer, how come that two women will be treated? (G2P6: 54, University Degree, false positive result)*  *And also, forty will have a false alarm but here it says, “ten of two hundred”. Forty along all the time… is that it?”* (G2P7: 54, University Degree, participates in the population-based screening programme, false positive).  *“I found it [decision aid] too long. It’s like it is from professionals and I do not think if this will reach women (…) I got lost, I would be more direct and use language much closer to women, this is still too scientific”* (Doctor in a cancer research centre). |
| Vahabi 2011^54^, Canada | Qualitative individual interview-based study | Iranian immigrant women | 50 | Thematic analysis | Iranian immigrant women have unique information needs influenced by historical, socio-political, and cultural experiences. Multimedia methods are preferred for communication. | *“Back home, some of my friends told me to never wear*  *tight bra because it can cause breast cancer. […] But I am*  *not sure if this is true. I guess this kind of information*  *would be helpful”* (age 61, some high school, in Canada  10 years).  *“Since we came to Canada our diet has changed. We cannot afford buying fresh food. They are very expensive. So we are relying more on frozen, canned and fast food. It would be helpful if we could get more information about what we should and should not eat*” (age 41, some university ⁄ college, in Canada 9 years). |
| Wiener 2018 ^55^, United States | Semi structured interviews and focus groups | Patients who had undergone lung cancer screening in prior year  Clinicians who refer patients for lung cancer screening | 49  36 | Direct content analysis | Clinicians had inconsistent information sharing and decision aid use, while patients receive limited information and lacked awareness. Barriers included time constraints, limited access to aids, and patient comprehension. | *“They sent you all this list of possibilities of what could happen, you’re thinking, ‘Holy Christ!”* (Patients).  *“There are these wonderful tools online, but when I’m sitting in front of a patient, I can never find them”* (Clinicians).  *“We don’t get very quantitative because...their eyes glaze over, and I’m not sure it has much meaning to them”* (Clinicians). |

COREQ= Consolidated Criteria for Reporting Qualitative Research

**References**

1. Allen, J. D. *et al.* A randomized trial of a computer-tailored decision aid to improve prostate cancer screening decisions: Results from the take the wheel trial. *Cancer Epidemiology Biomarkers and Prevention* **19**, 2172–2186 (2010).

2. Brenner, A. T. *et al.* Colorectal Cancer Screening in Vulnerable Patients: Promoting Informed and Shared Decisions. *Am J Prev Med* **51**, 454–462 (2016).

3. Cadet, T. *et al.* Evaluation of a mammography decision aid for women 75 and older at risk for lower health literacy in a pretest-posttest trial. *Patient Educ Couns* **104**, 2344–2350 (2021).

4. Gökce, M. I. *et al.* Informed decision making before prostate-specific antigen screening: Initial results using the American Cancer Society (ACS) Decision Aid (DA) among medically underserved men. *Cancer* **123**, 583–591 (2017).

5. Hoffman, A. S. *et al.* An entertainment-education colorectal cancer screening decision aid for African American patients: A randomized controlled trial. *Cancer* **123**, 1401–1408 (2017).

6. Hoffman, A. S. *et al.* Using a Patient Decision Aid Video to Assess Current and Former Smokers’ Values About the Harms and Benefits of Lung Cancer Screening With Low-Dose Computed Tomography. *MDM Policy Pract* **3**, (2018).

7. Housten, A. J. *et al.* Does Animation Improve Comprehension of Risk Information in Patients with Low Health Literacy? A Randomized Trial. *Medical Decision Making* **40**, 17–28 (2020).

8. Lau, Y. K. *et al.* Lung Cancer Screening Knowledge, Perceptions, and Decision Making Among African Americans in Detroit, Michigan. *Am J Prev Med* **60**, e1–e8 (2021).

9. Lepore, S. J. *et al.* Informed decision making about prostate cancer testing in predominantly immigrant black men: A randomized controlled trial. *Annals of Behavioral Medicine* **44**, 320–330 (2012).

10. Manners, D. *et al.* Development and evaluation of a consumer information resource, including Patient Decision Aid, for lung cancer screening: A quasi-experimental study. *Transl Behav Med* **10**, 404–412 (2020).

11. Miller, D. P. *et al.* Effectiveness of a web-based colorectal cancer screening patient decision aid: A randomized controlled trial in a mixed-literacy population. *Am J Prev Med* **40**, 608–615 (2011).

12. Miller, D. P. *et al.* Effect of a digital health intervention on receipt of colorectal cancer screening in vulnerable patients a randomized controlled trial. *Ann Intern Med* **168**, 550–557 (2018).

13. Perestelo-Perez, L. *et al.* Effectiveness of a decision aid for promoting colorectal cancer screening in Spain: A randomized trial. *BMC Med Inform Decis Mak* **19**, (2019).

14. Ruparel, M. *et al.* Impact of a lung cancer screening information film on informed decision-making: A randomized trial. *Ann Am Thorac Soc* **16**, 744–751 (2019).

15. Rubel, S. K. *et al.* Testing the effects of a decision aid for prostate cancer screening. *J Health Commun* **15**, 307–321 (2010).

16. Salkeld, G. *et al.* The role of personalised choice in decision support: A randomized controlled trial of an online decision aid for prostate cancer screening. *PLoS One* **11**, (2016).

17. Schapira, M. M. *et al.* The Impact of a Risk-Based Breast Cancer Screening Decision Aid on Initiation of Mammography Among Younger Women: Report of a Randomized Trial. *MDM Policy Pract* **4**, (2019).

18. Schroy, P. C. *et al.* The impact of a novel computer-based decision aid on shared decision making for colorectal cancer screening: A randomized trial. *Medical Decision Making* **31**, 93–107 (2011).

19. Schwartz, P. H. *et al.* Impact of including quantitative information in a decision aid for colorectal cancer screening: A randomized controlled trial. *Patient Educ Couns* **102**, 726–734 (2019).

20. Sferra, S. R. *et al.* Aiding shared decision making in lung cancer screening: Two decision tools. *Journal of Public Health (United Kingdom)* **43**, 673–680 (2021).

21. Smith, S. K. *et al.* A decision aid to support informed choices about bowel cancer screening among adults with low education: Randomised controlled trial. *BMJ (Online)* **341**, 977 (2010).

22. Volk, R. J. *et al.* Effect of a Patient Decision Aid on Lung Cancer Screening Decision-Making by Persons Who Smoke: A Randomized Clinical Trial. *JAMA Netw Open* **3**, (2020).

23. Williams, R. M. *et al.* Fostering informed decisions: A randomized controlled trial assessing the impact of a decision aid among men registered to undergo mass screening for prostate cancer. *Patient Educ Couns* **91**, 329–336 (2013).

24. Barry, M. J. *et al.* Responses to a decision aid on prostate cancer screening in primary care practices. *Am J Prev Med* **49**, 520–525 (2015).

25. Eden, K. B. *et al.* Mammography decision aid reduces decisional conflict for women in their forties considering screening. *J Womens Health* **24**, 1013–1020 (2015).

26. Halley, M. C., Rendle, K. A. S., Gillespie, K. A., Stanley, K. M. & Frosch, D. L. An exploratory mixed-methods crossover study comparing DVD- vs. Web-based patient decision support in three conditions: The importance of patient perspectives. *Health Expectations* **18**, 2880–2891 (2015).

27. Lau, Y. K. *et al.* Evaluation of a Personalized, Web-Based Decision Aid for Lung Cancer Screening. *Am J Prev Med* **49**, e125–e129 (2015).

28. Lewis, C. L. *et al.* A Randomized Controlled Effectiveness Trial for PSA Screening Decision Support Interventions in Two Primary Care Settings. *J Gen Intern Med* **30**, 810–816 (2015).

29. Lewis, C. L. *et al.* A Decision Aid to Promote Appropriate Colorectal Cancer Screening among Older Adults: A Randomized Controlled Trial. *Medical Decision Making* **38**, 614–624 (2018).

30. Reuland, D. S. *et al.* A pre-post study testing a lung cancer screening decision aid in primary care. *BMC Med Inform Decis Mak* **18**, (2018).

31. Sepucha, K. R. *et al.* Getting patients back for routine colorectal cancer screening: Randomized controlled trial of a shared decision-making intervention. *Cancer Med* (2022) doi:10.1002/cam4.5172.

32. Sheridan, S. L. *et al.* Shared decision making for prostate cancer screening: The results of a combined analysis of two practice-based randomized controlled trials. *BMC Med Inform Decis Mak* **12**, (2012).

33. Sheridan, S. L. *et al.* A comparative effectiveness trial of alternate formats for presenting benefits and harms information for low-value screening services a randomized clinical trial. *JAMA Intern Med* **176**, 31–40 (2016).

34. Taylor, K. L. *et al.* Decision making in prostate cancer screening using decision aids vs usual care a randomized clinical trial. *JAMA Intern Med* **173**, 1704–1712 (2013).

35. van Vugt, H. A. *et al.* Informed decision making on PSA testing for the detection of prostate cancer: An evaluation of a leaflet with risk indicator. *Eur J Cancer* **46**, 669–677 (2010).

36. Akanuwe, J. N. A., Black, S., Owen, S. & Siriwardena, A. N. Communicating cancer risk in the primary care consultation when using a cancer risk assessment tool: Qualitative study with service users and practitioners. *Health Expectations* **23**, 509–518 (2020).

37. Amélie, A. E. *et al.* What do women and healthcare professionals expect of decision aids for breast cancer screening? A qualitative study in France. *BMJ Open* **12**, (2022).

38. Baptista, S. *et al.* Translation and cultural adaptation of a prostate cancer screening decision aid: A qualitative study in Portugal. *BMJ Open* **10**, (2020).

39. Croes, K. D. *et al.* Core Elements of Shared Decision-making for Women Considering Breast Cancer Screening: Results of a Modified Delphi Survey. *J Gen Intern Med* **35**, 1668–1677 (2020).

40. Crothers, K. *et al.* Patients’ attitudes regarding lung cancer screening and decision aids: A survey and focus group study. *Annals of the American Thoracic Society* vol. 13 1992–2001 Preprint at https://doi.org/10.1513/AnnalsATS.201604-289OC (2016).

41. DuBenske, L., Ovsepyan, V., Little, T., Schrager, S. & Burnside, E. Preliminary Evaluation of a Breast Cancer Screening Shared Decision-Making Aid Utilized Within the Primary Care Clinical Encounter. *J Patient Exp* **8**, (2021).

42. Engelen, A., Vanderhaegen, J., Van Poppel, H. & Van Audenhove, C. The use of decision aids on early detection of prostate cancer: views of men and general practitioners. *Health Expectations* **20**, 221–231 (2017).

43. Friedman, D. B., Thomas, T. L., Owens, O. L. & Hébert, J. R. It Takes Two to Talk About Prostate Cancer: A Qualitative Assessment of African American Men’s and Women’s Cancer Communication Practices and Recommendations. *Am J Mens Health* **6**, 472–484 (2012).

44. Kuss, K., Adarkwah, C. C., Becker, M., Donner-Banzhoff, N. & Schloessler, K. Delivering the unexpected—Information needs for PSA screening from Men’s perspective: A qualitative study. *Health Expectations* **24**, 1403–1412 (2021).

45. Hernández-Leal, M. J. *et al.* Development of support material for health professionals who are implementing Shared Decision-making in breast cancer screening: Validation using the Delphi technique. *BMJ Open* **12**, (2022).

46. Maschke, A. *et al.* Discussions of Potential Mammography Benefits and Harms among Patients with Limited Health Literacy and Providers: “Oh, There are Harms?” *J Health Commun* **25**, 951–961 (2020).

47. Pannebakker, M. M., Mills, K., Johnson, M., Emery, J. D. & Walter, F. M. Understanding implementation and usefulness of electronic clinical decision support (eCDS) for melanoma in English primary care: A qualitative investigation. *BJGP Open* **3**, (2019).

48. Reese, T. J. *et al.* Implementing lung cancer screening in primary care: Needs assessment and implementation strategy design. *Transl Behav Med* **12**, 187–197 (2022).

49. Schapira, M. M. *et al.* How patients view lung cancer screening: The role of uncertainty in medical decision making. *Ann Am Thorac Soc* **13**, 1969–1976 (2016).

50. Schonberg, M. A. *et al.* Primary Care–Based Staff Ideas for Implementing a Mammography Decision Aid for Women 75+: a Qualitative Study. *J Gen Intern Med* **34**, 2414–2420 (2019).

51. Schwartz, P. H., O’Doherty, K. C., Bentley, C., Schmidt, K. K. & Burgess, M. M. Layperson Views about the Design and Evaluation of Decision Aids: A Public Deliberation. *Medical Decision Making* **41**, 527–539 (2021).

52. Tatari, C. R. *et al.* The SWIM study: Ethnic minority women’s ideas and preferences for a tailored intervention to promote national cancer screening programmes—A qualitative interview study. *Health Expectations* **24**, 1692–1700 (2021).

53. Toledo-Chávarri, A. *et al.* A qualitative study on a decision aid for breast cancer screening: Views from women and health professionals. *Eur J Cancer Care (Engl)* **26**, (2017).

54. Vahabi, M. Breast cancer and screening information needs and preferred communication medium among Iranian immigrant women in Toronto. *Health Soc Care Community* **19**, 626–635 (2011).

55. Wiener, R. S. *et al.* Patient and Clinician Perspectives on Shared Decision-making in Early Adopting Lung Cancer Screening Programs: a Qualitative Study. *J Gen Intern Med* **33**, 1035–1042 (2018).
